# An Update on Pharmacologic Management of Neonatal Hypotension: When, Why, and Which Medication

**DOI:** 10.3390/children11040490

**Published:** 2024-04-19

**Authors:** Eleni Agakidou, Ilias Chatziioannidis, Angeliki Kontou, Theodora Stathopoulou, William Chotas, Kosmas Sarafidis

**Affiliations:** 1 1st Department of Neonatology and Neonatal Intensive Care, Faculty of Medicine, School of Health Sciences, Aristotle University of Thessaloniki, Ippokrateion General Hospital, 54642 Thessaloniki, Greece; ihatzi@auth.gr (I.C.); angiekon2001@yahoo.gr (A.K.); dorastatho@gmail.com (T.S.); kosaraf@auth.gr (K.S.); 2 Department of Neonatology, University of Vermont, Burlington, VT 05405, USA

**Keywords:** blood pressure, catecholamines, inotropes, neonatal hypotension, neonates, pharmacology, preterm infants, anti-hypotensive agents, hydrocortisone

## Abstract

Anti-hypotensive treatment, which includes dopamine, dobutamine, epinephrine, norepinephrine, milrinone, vasopressin, terlipressin, levosimendan, and glucocorticoids, is a long-established intervention in neonates with arterial hypotension (AH). However, there are still gaps in knowledge and issues that need clarification. The main questions and challenges that neonatologists face relate to the reference ranges of arterial blood pressure in presumably healthy neonates in relation to gestational and postnatal age; the arterial blood pressure level that potentially affects perfusion of critical organs; the incorporation of targeted echocardiography and near-infrared spectroscopy for assessing heart function and cerebral perfusion in clinical practice; the indication, timing, and choice of medication for each individual patient; the limited randomized clinical trials in neonates with sometimes conflicting results; and the sparse data regarding the potential effect of early hypotension or anti-hypotensive medications on long-term neurodevelopment. In this review, after a short review of AH definitions used in neonates and existing data on pathophysiology of AH, we discuss currently available data on pharmacokinetic and hemodynamic effects, as well as the effectiveness and safety of anti-hypotensive medications in neonates. In addition, data on the comparisons between anti-hypotensive medications and current suggestions for the main indications of each medication are discussed.

## 1. Introduction

Arterial hypotension (AH) is a frequent problem in neonates with the potential to affect both short- and long-term outcomes. Clinical conditions associated with cardiovascular instability and low arterial pressure (AP) in preterm neonates include difficulties with adaptation to extrauterine circulation during the first 72 h after birth [1,2,3] and severe neonatal complications, such as sepsis, necrotizing enterocolitis (NEC), persistent pulmonary hypertension of the neonate (PPHN), perinatal asphyxia, congenital heart disease, and patent ductus arteriosus (PDA) [2,4,5,6]. Previous authors have outlined the controversies surrounding important issues, which include the lack of a generally accepted reference range of AP, the unclear definition of hypotension in neonates, and the effect of postnatal age on AP [7,8]. Furthermore, the complex pathophysiology of AH in preterm infants, either during the transition period or associated with complications of preterm birth, contribute to the existing controversies [9]. Another important challenge neonatologists face is the lack of generally accepted guidelines, which has led to variations between different neonatal centers regarding the frequency of anti-hypotensive treatment and the kind of medications used [4,10,11] Moreover, inotrope use may depend on the attending neonatologist’s discretion, which can further compromise the application of a generally accepted protocol for the management of AH in this vulnerable population. Another important issue is the conflicting results surrounding the effects of AH and its treatment with vasoactive medications on short-term outcomes. Data on long-term outcomes of preterm infants are insufficient to support definite conclusions [7,12,13,14,15,16,17,18,19,20].

In this review, after a brief reference to definitions of AH in neonates and available data on the pathophysiology of AH, we focus on the vasoactive medications used in neonates, including dopamine (DOP), dobutamine (DOB), epinephrine (EPI), norepinephrine (NE), milrinone (MIL), arginine vasopressin (AVP) and its derivative terlipressin, glucocorticoids, and the very rarely used in neonates levosimendan (LEVO). In addition, available data regarding the comparisons between the anti-hypotensive medications for their efficacy and safety, the potential association of AH and/or anti-hypotensive medications with long-term neurodevelopmental (ND) outcomes, and the main indications of each anti-hypotensive medication are discussed.

## 2. Method of Literature Search

Electronic databases (PubMed, Scopus, and the Cochrane Library) were searched up to January 2024 for articles on cardiovascular medications used in the treatment of neonatal hypotension. Moreover, a manual search of the reference lists of the included studies was conducted to find additional relevant articles. The MeSH terms used included “hypotension”, “vasoactive”, “hemodynamics”, “inotropic”, “vasopressors”, “catecholamines”, “dopamine”, “dobutamine”, “epinephrine”, “adrenaline”, norepinephrine”, “noradrenaline”, “milrinone”, “vasopressin”, “terlipressin”, “levosimendan”, “corticosteroids”, “hydrocortisone”, “dexamethasone”, “infant”, “neonate”, “randomized controlled trials”, “review”, “systematic review”, “placebo”, “drug therapy”. Studies included full reports in English language. Arterial hypotension was accepted as defined in individual studies.

## 3. Definitions of Arterial Hypotension in Neonates

Thus far, there is no standard definition of AH in neonates. The definitions being used in clinical practice and research are mostly based on gestational age (GA) [21]. Some studies defined AH as the mean AP less than the mean or 5th percentile of GA-related reference values [21]. However, the reference ranges of AP defined after the GA and postnatal age vary widely [7]. Other definitions of hypotension assume that a mean AP below 30 mm Hg is associated with compromised brain perfusion in neonates with a birth weight of less than 1000 g, i.e., extremely low birth weight infants (ELBWI). This definition of AH suggests the need of anti-hypotensive treatment in all preterm infants with AP below this value, without consideration for gestational or postnatal age [9]. Finally, the definition that is most often used is based on the AP range reported by the Joint Working Group of the British Association of Perinatal Medicine (BAPM), which recommended that the mean AP should be kept at or over the GA in weeks [22]. Likewise, the German Neonatal Network (GNN) reported that the lowest limits of mean AP on day one were similar to the GA in weeks [23]. Inconsistencies in the suggested reference ranges of early AP, and consequently among definitions of AH in preterm infants, are attributed to methodological limitations of the relevant studies, which begs the question of the reliability and usefulness of the proposed lower “normal” values as a criterion of AH [7]. Besides the GA, the progressive increase in AP after the first few days of life should be considered when defining ‘normal’ AP in preterm infants [8].

Within the context of the limitations of using AP as the exclusive criterion for defining AH, clinical and laboratory surrogate markers of cardiovascular failure have been suggested, including capillary refill time, base excess, and blood lactate as indicators of poor tissue perfusion [24]. Moreover, the use of targeted neonatal echocardiography and Near-InfraRed Spectroscopy (NIRS) monitoring for assessing heart function and cerebral perfusion, respectively, can add to the identification of AH needing inotrope/vasopressor treatment. The importance of NIRS as a complementary tool for the evaluation of the hypotensive neonate is supported by studies showing that cerebral oxygen delivery may not correlate with the presence of hypotension [20,25]. This finding suggests that the low AP measurement cannot accurately indicate potentially decreased cerebral oxygenation, which is a central target of anti-hypotensive treatment [20,25,26,27].

## 4. Pathophysiology of Hemodynamic Instability in Neonates

Hemodynamic instability is characterized by the compromised function of organs due to alterations of the cardiovascular system, decreased oxygen transportation, and impaired autoregulation of organ blood flow [5,25]. It is more frequent in preterm neonates due to the developmental immaturities of their cardiovascular system [28,29]. Compared to adults, the immature myocardium of preterm neonates contains a lower proportion of contractile fibers (30% in infants versus 60% in adult myocardium), which are less organized [30,31]. In addition, neonatal myocardium contains more water and has an immature sarcoplasmic reticulum highly dependent on extracellular calcium. Normally, neonatal myocardium displays increased basal contractility functioning near its physiological ability. Consequently, neonatal myocardium is not capable of adapting adequately to hemodynamic changes at birth [1,2,3,32] or in the presence of complications, such as perinatal hypoxia, PPHN, and sepsis [4,5,6,32,33]. This maladaptation is especially important during the transition from fetal to neonatal circulation, when the low vascular resistance of the placenta is replaced by the higher vascular resistance of neonatal circulation [5,29,34,35]. Furthermore, the increased pulmonary vascular resistance in fetal life may remain elevated after birth leading to PPHN that further compromises the adaptation to extrauterine conditions [32].

The presence of a hemodynamically significant PDA is also an important contributor to the hemodynamic instability of preterm neonates [35,36,37]. It decreases diastolic AP and impairs myocardial perfusion with subsequent myocardial dysfunction and left-to-right shunting, eventually limiting peripheral perfusion [38,39]. More recently in a case control study, Aldana-Aguirre et al. performed a comprehensive echocardiography in a highly selective group of non-treated hypotensive ELBWI during the first day of life [5]. It was found that, compared to matched normotensive controls, the hypotensive infants had low systemic afterload and increased prevalence and size of PDA, without left ventricular dysfunction. These findings suggest that isolated hypotension during the immediate postnatal period in ELBWI may be attributed to either reduced systemic vascular resistance (SVR) or a large PDA [40,41].

Besides immaturity and the presence of a PDA, additional factors potentially affecting the hemodynamic status of the neonate include the time elapsed after birth; neonatal complications, including sepsis, NEC, air leak syndromes, severe respiratory distress syndrome, pulmonary hemorrhage, cerebral hemorrhage, and ductal-dependent congenital heart disease; as well as interventions, such as mechanical ventilation, or drug administration, such as sedation [32]. In fact, Burns et al. showed that the use of inotropes was notably more common in neonates with NEC (72.4%), PPHN (42.1%), and in those with GA less than 28 weeks (28.2%). Other situations associated with inotropic treatment initiated in the first week of life included birth asphyxia with pulmonary hypertension, neonatal surgery, neonatal sepsis, and congenital heart disease [4].

Another cause of hemodynamic derangement, concerning mainly very low birth weight infants (VLBWI), late preterm, and asphyxiated neonates, is the relative primary or secondary adrenal insufficiency, low cortisol levels, and decreased vascular responsiveness to catecholamines partly attributable to the downregulation/desensitization of cardiovascular adrenergic receptors [42,43,44,45,46]. The relative or absolute adrenal insufficiency in preterm infants reduces the vascular response to catecholamines, eventually leading to catecholamine-resistant shock that improves following corticosteroid administration [46,47].

The vascular tone can also be affected by endocrine, paracrine, and other factors of the autonomic nervous system, such as endothelin, AVP, and nitric oxide due to their effects on vascular tone. In this respect, pathological conditions inducing a systematic inflammatory response syndrome, such as sepsis and NEC, may cause vasodilation secondary to the disruption of nitric oxide (NO) production by the vascular endothelium [30,32,48].

In summary, existing pathophysiological data suggest that multiple and complex mechanisms underlie the development of AH in the neonatal period and underline the importance of a strong understanding of the pathophysiology of each neonatal situation associated with AH in order to design the best management approach.

## 5. Anti-Hypotensive Medications Used in Neonates

Management of AH must be based on the underlying pathology as indicated by the perinatal–neonatal history and clinical presentation. Interventions include volume expansion, vasoactive medications, and corticosteroids. Volume expansion with intravenous colloids or crystalloids is administered initially in preterm infants in 85% of neonatal intensive care units (NICUs), followed by anti-hypotensive agents [49]. Medications used for AH in neonates include vasoactive agents (inotropes and vasopressors) and corticosteroids [9,50,51,52].

Vasoactive medications are classified as (a) vasopressors, which affect vascular tone and are further subclassified into vasoconstrictors and vasodilators, and (b) inotropes, which are subclassified into those possessing positive or negative inotropic actions on the heart [28,53]. Furthermore, vasoconstrictors are subclassified into pure vasoconstrictors (phenylephrine and AVP) and inoconstrictors (DOP, EPI, and NE). Inotropes include inodilators (DOB and MIL) and the above-mentioned inoconstrictors [53,54]. Inotropes’ main function is to improve myocardial contractility leading to increased cardiac output, while vasopressors increase AP by inducing peripheral vasoconstriction [28]. Of note, certain vasoactive medications, such as DOP, EPI, and NE, act via stimulation of alpha- and beta-adrenergic receptors, as well as dopaminergic receptors depending on dosage, thereby exerting both inotropic and vasopressor effects [50,53]. The characteristics of hypotensive medications used in neonates are summarized in Table 1. Factors that may influence the effect of inotropes include the degree of immaturity, cardiovascular function, and the pathophysiology and severity of underlying diseases [9,55].

### 5.1. Dopamine

**Figure 1:**
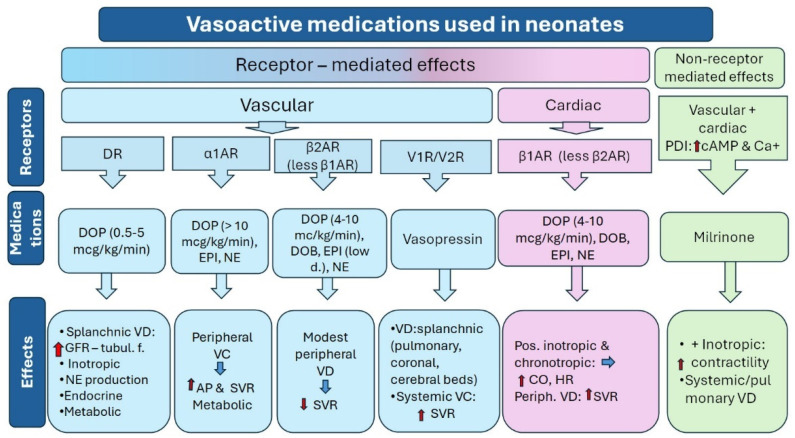
Summary of the effects of the vasoactive medications used in neonates. AP, arterial pressure, AR, adrenoreceptors; CO, cardiac output; DR, dopaminergic rec; HR, heart rate; PDI, phosphodiesterase inhibitor; SVR, systemic vascular resistance, tubul; f, tubular function; VC, vasoconstriction; VD, vasodilation; V1R/V2R, vasopressin receptors 1/2.

Dopamine (3-hydroxytyramine) is an endogenous catecholamine that is widely distributed within human organs and tissues (central nervous system, plasma, and other tissues) [50,53,54,99]. It is a key neurotransmitter of the central nervous system and peripheral organs, acting on different cells through alpha- and beta-adrenergic receptors and dopaminergic receptors. In this frame, DOP regulates crucial functions of the central nervous system (movement, cognition, reward, learning) and peripheral organs (cardiovascular function, intestinal motility, sodium levels, hormone release, and immune functions) [100]. DOP exerts its actions via stimulation of dopaminergic (D1–D5) and adrenergic (alpha-1, beta-1, and beta-2) receptors (Figure 1). It induces arterial vasoconstriction and increases cardiac output leading to an increase in AP [101]. In addition to the direct effects on the cardiovascular system, DOP is a precursor of NE and EPI, which are byproducts of the DOP metabolic pathway. It is most frequently used in neonates anti-hypotensive medication with a prescription rate ranging between 65.3 and 83% [49,100,102].

Early pharmacokinetic studies show that plasma concentrations of DOP varied widely among individuals and were not correlated with AP. An early study in children aged from 3 months to 13 years recovering from cardiac surgery showed wide individual variations in pharmacokinetic variables, regardless of clinical stability. DOP pharmacokinetics were not affected by liver or renal dysfunction [103]. The elimination half-life was 26+/− 14 (SD) min with a clearance rate linearly related to dose only with DOB co-administration [103]. However, other authors have reported that serum levels of DOP in stable neonates are proportionally related to the dose given [104]. A potential explanation for the variation in DOP levels suggested by previous authors in the late 1980s and early 1990s may be the low endogenous NE level in severely ill patients that is replaced by different proportions of the administered DOP [105]. Specifically, a study in 11 severely ill infants with sepsis and hypotensive shock receiving DOP infusion showed that at steady state, plasma DOP levels ranged between 0.013 and 0.3 mcg/mL and the elimination half-life was 6.9 min. No significant correlation between DOP pharmacokinetics and GA, birth weight, or postnatal age was found. Moreover, the high inter-individual variations in AP response to DOP infusion observed in sick infants could reflect differences in pharmacokinetics, plasma concentrations, or underlying pathophysiology [105]. These findings suggest that the effectiveness of DOP can only be evaluated by the clinical response rather than predicted by the dosage used [105].

DOP is the most widely studied inotrope [49,102]. Several observational studies in preterm infants showed that DOP at doses of 5 mcg/kg/min or 10 mcg/kg/min are effective in increasing the AP secondary to increased cardiac output and arterial vasoconstriction [101,106]. The reported individual differences in the cardiovascular response to DOP in preterm neonates, especially those critically ill, can be partly attributed to differences in the expression and function of the cardiovascular adrenergic receptors. The receptor activity may also be affected by adrenal insufficiency, dysregulation of locally produced vasodilators, such as prostaglandins and nitric oxide, or disease severity [57,106]. The effect of DOP on AP and various organs is dose-dependent, likely due to differences in plasma concentrations, disease pathophysiology, or disequilibrium of its action on the peripheral vessels and heart [9,40,56]. At low doses (0.5 to 2 mcg/kg/min), it mainly stimulates the dopaminergic receptors, which cause vasodilation in renal, mesenteric, and coronary vascular beds [40,51,57]. At usual doses (infusion at 2–10 mcg/kg/min), DOP exerts dopaminergic and alpha-1 vasopressive effects (at doses of 2–5 mcg/kg/min) and beta-1/beta-2 inotropic effects (at doses 4–10 mcg/kg/min) via stimulation of the respective adrenergic and dopaminergic receptors of myocardium [40,54,101]. Overall, stimulation of the adrenergic and dopaminergic receptors increases HR, cardiac contractility, AP, and cerebral blood flow in hypotensive neonates [40,54,101]. Moreover, stimulation of dopaminergic receptors exerts multiple actions including endocrine effects, increase in lung fluid clearance, glomerular filtration rate (GFR), and tubular functions [40,52,107]. At high doses (≥10 mcg/kg/min), DOP stimulates the vascular alpha-1-adrenergic receptors causing vasoconstriction that leads to increases in systemic, and possibly pulmonary vascular resistance [9,40,56,108]. Data indicate that at high doses, the vasopressor properties of DOP predominate over the inotropic actions, leading to increased myocardial contractility and cardiac output [16]. However, a potential imbalance between inotrope and vasopressor actions of DOP at high doses may increase vasoconstriction with a subsequent decrease in cardiac output [56,58,108]. The vasoconstrictive effect of DOP is more pronounced in infants with PPHN, in whom high doses may have an unpredictable effect and may further increase the pulmonary vascular resistance, leading to deterioration of the right-to-left shunting via the PDA and subsequent hypoxia [50,51]. Administration of even higher doses of DOP (>20 mcg/kg/min) to preterm infants raises concern for the potentially excessive peripheral vasoconstriction and the consequent reduction in cardiac output [56]. Although there is no evidence that high-dose DOP has detrimental vasoconstrictive effects, clinicians prefer to add DOB rather than further increase the dose of DOP [40]. Another issue concerning DOP administration regards how long the medication retains its activity in infusion fluids and, consequently, the frequency of DOP infusion changeover. A study by Kirupakaran et al. could not prove any changes in DOP concentration in the infusion fluids over 12 h, while the initial DOP concentration decreased by up to 15% after 24 h. The authors suggested that changing infusions every 12 h reduces the mean AP fluctuations [109].

In addition to its direct vasoactive effect, DOP induces the release of NE, which accounts for approximately 30–50% of DOP inotropic action on the myocardium [57,106].

Most studies looking at the hemodynamic effects of DOP in preterm infants have been performed during the transition period, while studies performed later are limited [9,16,40]. Consequently, the data reported by these studies should only cautiously be extrapolated to preterm neonates with higher postnatal age, especially in the presence of other morbidities.

Assessment of cerebral perfusion in VLBWI using NIRS showed that DOP may have a negative effect on cerebral vessel autoregulation [110]. A study in 28 VLBWI (GA < 30 weeks) treated for hypotension during the first 3 days of life reported that increasing AP may not increase cerebral oxygenation [111]. The lack of association with cerebral oxygenation may be attributed to the fact that many VLBWI are able to autoregulate their cerebral blood flow [111]. Moreover, treatment with inotropes may further impair cerebral autoregulation in preterm infants [110]. Nevertheless, this effect had not been reported by preceding studies, including a meta-analysis of observational studies, which showed that DOP increases both AP and cerebral perfusion [107,112]. A study by Osborn et al. indicated that DOP did not adversely affect the combined rate of death and/or ND delay at the corrected age of 3 years [60]. Another important side effect of DOP is derived from its regulating actions on hormone production, which includes a transient decrease in the production of thyroid-stimulating hormone (TSH), growth hormone, and prolactin [57]. The inhibitory effect on thyroid-stimulating hormone production could change the results of neonatal screening. Therefore, it is suggested that the measurement should be repeated at least 12 h after the interruption or discontinuation of DOP. Other adverse effects of DOP are related to the potentially excessive peripheral vasoconstriction at higher doses, which may decrease cardiac output [57,113]. Finally, an early retrospective case series analysis of two groups of 41 consecutive high-risk ELBWI demonstrated an increased risk of retinopathy of prematurity in infants treated with DOP compared to the non-DOP-treated group [114].

### 5.2. Dobutamine

Dobutamine hydrochloride is a synthetic catecholamine that exerts its actions via stimulation of predominantly beta-1 adrenergic receptors. It increases myocardial contractility, heart rate (HR), and cardiac output (Table 1) [50]. Moreover, clinical and experimental studies showed that DOB produces moderate vasodilation via binding to peripheral beta-2 receptors [9,113]. DOB is the second most used first-line anti-hypotensive medication for neonatal hypotension following DOP [49]. It is also used as a second-line medication, or combined with DOP when the latter medication fails [49]. The recommended dosage of DOB lies between 5 and 20 mcg/kg/min via continuous infusion [63]. However, despite children receiving similar dosages, a wide variation in serum concentrations of DOB has been observed [9].

Several pharmacodynamic and pharmacokinetic studies have been performed in children, but data in preterm neonates are limited and largely conflicting [63]. Early pharmacodynamic studies in children demonstrated that DOB infusion increased AP and decreased pulmonary capillary mean blood pressure, but regardless of dosage, it had no effect on other hemodynamic parameters, such as HR, pulmonary and right atrial mean pressure, and systemic and pulmonary vascular resistance [62]. In contrast, later studies in neonates and children showed that DOB infusion increased HR, left and right ventricular output, and systemic mean AP via actions on beta-1 adrenergic receptors [63,115,116]. Studies assessing the effect of DOB on pulmonary vascular resistance in children reported contradictory results [63]. It was found that DOB infusion at a rate of 2.5 to 10 mcg/kg/min did not affect [62], or increase [117] or decrease [118], pulmonary vascular resistance. Although this effect was not investigated in neonates, neonatal studies showed that DOB infusion decreased requirements of inspired oxygen, which indicate that DOB may decrease pulmonary vascular resistance [63,119,120]. Contradictory results were also reported for the effect of DOB on systemic vascular resistance in preterm neonates. Most studies suggested a decreasing effect [63,119], while other authors found that doses up to 10 mcg/kg/min caused a non-significant increase in systemic vascular resistance [56]. Hemodynamic effects of DOB reported in preterm infants include improvement in myocardial performance index, increase in cardiac output, and improved blood flow in the superior vena cava (SVC), as well as the cerebral, mesenteric and renal arteries [56,63,119]. A review of pharmacokinetic studies by Mahoney et al. confirmed that serum levels of DOB were positively associated with the infusion rates, but they varied widely between patients receiving similar doses [63]. Two recent studies assessed the pharmacokinetics and/or pharmacodynamics of DOB during the transitional period. It was found that the pharmacokinetics followed a one-compartmental linear model and that clearance correlated with postmenstrual age and birth weight [14,121]. Moreover, plasma levels were related with hemodynamic parameters, including right ventricular output, left ventricular ejection fraction (linear model), left ventricular output, HR, and mean AP (sigmoidal E_max_ model) [63,64,121].

Overall, pharmacokinetic studies have reported varying models of plasma DOB clearance including a negative correlation with infusion rate, a simple log linear decline in plasma concentration, and a biphasic decline suggestive of a two-compartment model. The wide inter- and intra-individual variation reported by hemodynamic and pharmacokinetic data requires awareness and underlines the need for individualized DOB dosage [63,121]. A review of pharmacokinetic studies of DOB in neonates by Mahoney et al. synopsized the results as follows: (a) the infusion rate was positively correlated with plasma levels; (b) there was a great interindividual variation concerning the elimination kinetics of DOB clearance; (c) the lack of homogeneity in the design, the wide range of the study population GA and age, underlying disease, and DOB dosage do not allow a reliable interpretation of the reported findings [63].

The most commonly reported adverse effects of DOB include tachycardia, hypotension, and ventricular arrhythmias, which usually occur with doses higher than 7.5 mcg/kg/min [63]. Follow-up studies at 3 and 6 years of life have shown conflicting results regarding its long-term effects on neurodevelopmental outcome [14,60].

### 5.3. Epinephrine

Epinephrine, or adrenaline, is an endogenous catecholamine that acts via the alpha- and beta-adrenergic receptors [40,51]. The hemodynamic effects on circulation are dose dependent. At a low dose of 0.02–0.1 mcg/kg/min, it stimulates the alpha-2, beta-1, and beta-2 receptors causing vasodilation in the systemic and pulmonary circulations, as well as increased heart contractility and HR, eventually leading to increases in AP and cerebral blood flow [40,51]. At higher doses (>0.5 mcg/kg/min), the alpha-1-mediated vasoconstrictive effects of EPI predominate, causing vasoconstriction and increased HR [40,51]. Blood flow to the gut and kidneys decreases, while heart and cerebral blood flow and tissue oxygen consumption increase [40,66]. Local ischemia secondary to alpha-1 vasoconstriction and beta-2 receptor-mediated induction of the anaerobic glycolytic pathway can lead to metabolic derangement [122].

In clinical practice, EPI is administered via continuous infusion at doses ranging between 0.05 and 1.0 mcg/kg/min [9]. Very high doses of EPI (>1 mcg/kg/min) are not recommended as they may increase mortality in preterm infants [9,123]. Nevertheless, treatment with EPI for neonatal shock is considered safe and effective [65]. It is usually administered as a third-line anti-hypotensive medication in neonates not responding to DOP and DOB [9]. In addition, surveys of clinical practice revealed that EPI is used in some settings as a first-line anti-hypotensive treatment [9,49].

Pharmacokinetic studies of exogenous EPI in critically ill children showed that during steady-state infusions, the plasma concentrations, but not clearance rate, were linearly related to the dose [124]. Pharmacodynamic studies in neonates subjected to cardiac surgery showed that EPI clearance, degree of HR changes, and AP were proportionally related to BW [51,125]. This relationship is attributed to immaturity of the neonatal myocardium, especially in VLBWI, along with age-related beta-1 and beta-2 receptor density [51,125].

Studies on EPI administration to hypotensive neonates are very limited, especially when considering preterm neonates [51,65,126]. An early retrospective study involving 31 VLBWI (GA 23–30 weeks), who did not respond to DOP infusion up to 15 mcg/kg/min, showed that EPI infusion at doses ranging from 0.05 and 2.6 mcg/kg/min significantly increased mean AP and HR, with a parallel increase in the base deficit [67]. In this line are the results of a recent retrospective cohort study in 115 term and preterm infants with hypotension. It was found that continuous infusion of EPI at low doses (<0.05 mcg/kg/min) resulted in a significant improvement in AP and urine output in 55% of the patients, without adverse effects [126].

Adverse effects of EPI that have been reported in neonates receiving high doses include tachycardia, hyperglycemia, tissue necrosis due to EPI extravasation, and increased peripheral vascular resistance, potentially leading to decreased tissue perfusion with a subsequent increase in plasma lactate levels [65,66,106].

### 5.4. Norepinephrine

Norepinephrine is a hydroxylated derivative of the DOP metabolic pathway which stimulates alpha- and beta-receptors to exert inotropic and peripheral vasoconstrictive effects [50,51]. It is a potent alpha-1 agonist, with a moderate to weak effect on beta-1 and beta-2 adrenergic receptors [53,101]. Pharmacodynamic studies in adults showed that NE increases systemic AP and may contribute to the decrease in pulmonary hypertension, thereby improving cardiac output and venous return via activation of beta-1 receptors [53]. A pharmacokinetic study in a pediatric population of 39 children, aged 0.1 to 189 (mean age 3.9) months (including 11 neonates of which 7 were preterm), showed that NE kinetics are described by a one-compartment linear model with linear elimination. The main factors impacting NE clearance and endogenous production rate were the bodyweight and age [125].

NE is administered at an initial dose of 0.01–0.04 mcg/kg/min (usual range 0.01–0.3 mg/kg/min) titrated by 0.02–0.04 mcg/kg/min every 5–15 min until achievement of the desired hemodynamic effect [53]. It has been reported that in cases of excessive vasodilatation and right ventricular failure requiring high doses of EPI (>0.1–0.2 mcg/kg/min), the addition of NE at doses of 0.02–0.05 mcg/kg/min facilitates de-escalation of EPI, thereby decreasing the probability of side effects [51,53]. However, the risk of vasoconstriction at high doses, which could adversely affect cardiac output and tissue perfusion, has limited the use of NE in neonates [51,127,128]. Therefore, NE doses higher than 0.5 to 1 mcg/kg/min are not recommended [68,71,72].

Published studies thus far are mainly retrospective, focusing predominantly on the effectiveness of NE in treating neonates with septic shock non-responsive to other inotropes and/or PPHN [68,69,70,71,129]. A prospective observational study of 22 late preterm and term neonates with refractory septic shock demonstrated that NE increased systemic AP and urine output, and decreased blood lactate levels, indicating an improvement in cardiac function and tissue perfusion [72]. The effect of NE on AP in preterm and term infants with septic shock, pulmonary hypertension, or isolated systemic hypotension was confirmed by several retrospective studies which showed significant improvement in urine output, arterial blood gas values, and tissue perfusion, with decreased requirements for oxygen and inotrope support [68,70,71,129,130]. The beneficial effect of NE on shock refractory to DOP and/or DOB is attributed to its combined inotropic and pulmonary vasodilatory effects [129,131]. In clinical practice, NE is traditionally used as a second- or third-line therapy or as adjuvant anti-hypotensive medication [68,70,71,132]. In addition, NE has been recommended as first-line inotrope in adults and neonates with severe shock, either septic or cardiogenic, or of non-identified origin, characterized by low systematic vascular resistance, instead of the traditional DOP/DOB combination [54,69,133,134]. Tachycardia is the most common side effect reported in about 30% of treated infants [68,70,71,132].

### 5.5. Milrinone

Milrinone is a phosphodiesterase type III inhibitor that acts on the myocardium by increasing intracellular cyclic AMP and calcium concentrations via inhibition of cAMP degradation [51,73,135]. It exerts positive inotropic and lusitropic effects on myocardium while decreasing systemic and pulmonary vascular resistance [136]. The vasodilatory activity is not affected by beta-blockers since it is not directly beta-1-receptor-mediated [137,138]. These properties make MIL a medication of choice for patients with low cardiac output and increased peripheral resistance and in neonates with PPHN [73,139,140,141]. However, the risk of severe hypotension should be always considered [73,74,142].

Several studies have assessed the pharmacokinetics of MIL in term and preterm neonates receiving MIL for PPHN and/or congenital heart disease [74,142]. A prospective two-stage open-labelled, dose-escalation pharmacokinetic study in preterm infants with GA < 29 weeks and chronological age less than 24 h documented a median half-life of 1.47 h (range, 0.62 to 10.85 h) [75]. The recommended therapeutic dosage of MIL was based on the dosage suggested for the pediatric population and results from neonatal pharmacokinetic studies [75]. Based on previous studies and their own research data, Paradisis et al. conducted a simulation model of MIL concentration–time data to achieve a target concentration of 180 to 300 ng/mL. The infusion regime that best fitted this target was a loading infusion of 0.75 mcg/kg/min for 3 h, followed by a maintenance infusion of 0.2 mcg/kg/min until 18 h of age [75]. A similar dosing regimen was recommended 10 years later by Hallik et al., who performed a pharmacokinetic study involving 31 neonates with a mean GA of 26 weeks and postnatal age of 13 days. The authors recommended a bolus infusion of 0.73 mcg/kg/min over 3 h, followed by a continuous infusion of 0.16 mcg/kg/min in preterm neonates [9,76]. In clinical practice, the dosing regimens of MIL may vary, but the most often used loading dose is 50 mcg/kg, followed by a continuous infusion for maintenance [9]. Of note, the dosage needs to be adjusted for renal function, prematurity, and chronologic age in order to decrease the possibility of accumulation and/or side effects [74,77,143].

Clinical studies on the use of MIL in neonates mainly refer to the treatment of PPHN, post-operatively following corrective cardiac surgery, and the prevention and treatment of low left ventricular output syndrome following PDA ligation [144]. Early studies by Paradisis et al. showed that MIL did not prevent the occurrence of low cardiac output syndrome (LCOS) in high-risk preterm neonates [145]. In contrast, several studies and case reports provided evidence that prophylactic MIL administration after PDA ligation may decrease hemodynamic derangement postoperatively [143,144,146,147]. It was found that MIL administration to preterm infants with PPHN, or after PDA ligation, resulted in recovery of left ventricular performance [147], increased cardiac troponin [147], decreased inotrope requirement, lower occurrence of ventilation failure [146], improvement in cardiorespiratory parameters (oxygenation index, dose of inhaled nitric oxide, and fraction of inspired oxygen), and echocardiographic indicators of myocardial performance and pulmonary hypertension [143,146]. The above studies indicate that treatment with MIL possibly has a beneficial effect on cardiovascular instability or in-hospital outcomes. However, these effects of MIL were not confirmed by a more recent retrospective study [136]. A Cochrane review investigated the effectiveness of postoperative prophylactic MIL administration to children (0 to 12 years of age) undergoing cardiac surgery in preventing LCOS and mortality. The authors concluded that the available evidence is insufficient to support or reject any significant effect of MIL in preventing LCOS or death [148].

Regarding the side effects, the metanalysis and most clinical studies did not show an increased risk of side effects in patients treated prophylactically with MIL [145,148,149,150]. A study reported that the most common side effects were thrombocytopenia and hypotension requiring vasopressors, which suggest a cautious use of MIL in neonates [77].

In summary, published findings concerning the effectiveness of MIL in preventing LCOS, decreasing hemodynamic dysfunction in preterm infants developing PPHN, or at the perioperative period post-PDA ligation are controversial. Moreover, rare but important side effects include thrombocytopenia and hypotension.

#### Vasopressin and Terlipressin

Vasopressin, or arginine vasopressin (AVP), or antidiuretic hormone (ADH), is a nonapeptide synthesized as a prohormone, primarily in hypothalamic neurons. It plays a key role in the control of blood pressure, osmotic balance, kidney function, and sodium homeostasis [151,152]. Three types of AVP receptors have been identified: V1a, V1b (also called V3), and V2. V1a receptors, expressed in vascular smooth muscle and the central nervous system, induce vasoconstriction, and control many brain functions. V2 receptors are predominantly expressed in kidneys and regulate water reabsorption [153]. V1b receptors are expressed in the brain, where they stimulate ACTH secretion [154,155]. AVP, administered at low doses, stimulates V2 receptors, causing selective vasodilation of coronary, pulmonary, and cerebral vessels. Furthermore, it stimulates V1a receptors, which induces vasoconstriction of other peripheral vessels. Furthermore, stimulation of V2 receptors in the kidney increases reabsorption of free water [154,156]. Combined, these mechanisms lead to increased systemic AP and decreased pulmonary pressure in infants with pulmonary hypertension [157].

Due to its short half-life (5 to 45 min), AVP is administered via continuous infusion [9,82,158,159]. The doses used in previous studies vary widely between 0.00001 and 0.003 U/kg/min [51,80]. Higher doses should be avoided since they have been associated with reductions in cardiac output and oxygen delivery, which may impair tissue perfusion and induce ischemic tissue injury [78,80,82,157,160,161]. Of note, different measurement units of AVP have been reported in the literature, making comparisons between studies difficult [162].

Published clinical studies are mostly retrospective and assess the effectiveness of AVP in restoring catecholamine-refractory hypotension and reducing pulmonary arterial hypertension [157,160,161,163,164]. They have been predominantly performed in neonates with catecholamine-resistant shock [80,82,161], septic shock [80], PPHN, and congenital diaphragmatic hernia [157], or following operation for congenital heart disease [160]. Recommendations of the Surviving Sepsis Campaign suggest administration of vasopressin in second line after norepinephrine, to maintain mean blood pressure goal or to decrease norepinephrine dosage [133].

Studies published more than 5 years ago showed that AVP increased AP while decreasing the pulmonary–systemic pressure ratio, HR, and the need for supplementary oxygen. Moreover, AVP improved left ventricular function (in about 50% of the patients) and the oxygenation index, thereby decreasing the need for extracorporeal membrane oxygenation. Other studies have reported that neonates treated with AVP required less fluid resuscitation and had decreased inotropic scores [82,160,161,163]. Also decreased was the duration of mechanical ventilation and length of stay in cardiovascular intensive care unit. No adverse effects on pulmonary hemodynamics were observed [80,82,160,161].

More recent clinical studies confirmed the efficacy of AVP as a rescue treatment in neonates with PPHN and catecholamine refractory hypotension [79,85,164,165,166,167,168]. Common findings of these studies were the increase in AP and improvement in oxygenation and requirements for inotropes following the addition of AVP to the treatment regime. Additional findings included reduction in lactic acidosis [79], avoidance of extracorporeal membrane oxygenation in 50% of the patients [164], and improved NIRS findings [167].

Terlipressin (triglycyl–lysine vasopressin) is a non-selective synthetic analogue of AVP acting via stimulation of V1 (vascular), V2 (renal), and V3 (pituitary) receptors [83,154,155]. In the circulation, it is converted to lysine AVP that is slowly released. Terlipressin has hemodynamic actions comparable to those of AVP, albeit with a different pharmacokinetic profile. It has a higher affinity to V1 receptors, and a longer half-life (6 h versus 6 min), which leads to an AP increase lasting for about 5 h. Early pharmacokinetic studies of terlipressin in adult volunteers suggested an intermittent intravenous bolus dose of 5, 10, or 20 mcg/kg every 4 to 6 h [83,86,159,168]. The reported doses given to neonates with refractory hypotension were 5–20 mcg/kg bolus, followed by maintenance doses of 5–20 mcg/kg every 4 h [169].

Data on terlipressin use in neonates are limited to case reports. Our search in PubMed revealed that only 13 cases of neonates with volume and catecholamine–refractory hypotension treated with terlipressin have been reported thus far. Terlipressin effectively restored AP in 12/13 neonates, improved tissue perfusion, and decreased the use of catecholamines [80,83,84,85,86,87,88,89,90,91]. The all-cause mortality rate was 46% (6/13). No adverse effects were reported, while ND outcome in two survivors, who were followed up, was normal [83,84,86,87,88,89,90,91,170].

Several systematic reviews and meta-analyses have evaluated the effectiveness of AVP and/or terlipressin as rescue or adjuvant therapy of fluid- and catecholamine–refractory shock in mixed pediatric/neonatal populations. It was reported that AVP or terlipressin administration resulted in a significant increase in AP, with a reduction in the inotropic score, HR, and serum lactate levels, along with improved end-organ perfusion and urine output [83,158,171]. On the other hand, no significant beneficial effect of either medication, in terms of mortality, length of stay in the intensive care unit, or tissue ischemia was found [83,158,171].

Multiple adverse effects of AVP and terlipressin have been reported, including increased plasma levels of liver enzymes, transient thrombocytopenia, hyponatremia, mitral regurgitation, gastric perforation, rhabdomyolysis, skin and limp ischemia, and hepatic necrosis on autopsy (n = 1) [81,83,155,157,171,172]. Although hyponatremia was not confirmed by other studies, close monitoring is suggested [161]. Moreover, the reported association between high doses of AVP and terlipressin with reductions in cardiac output and oxygen delivery lead to the recommendation of avoiding high doses [87,172,173,174,175]. Considering the possibility of these concerning side effects, it is recommended that the doses of AVP and terlipressin in neonates do not exceed the suggested maximum doses of 0.03 (or 0.067) U/min and 2 mcg/kg/h, respectively, while blood electrolytes, liver enzymes, and platelets should be closely monitored [81,82,83,155,157,164,171].

In summary, available data regarding the role of AVP and its homolog terlipressin as a rescue therapy in neonates with volume and catecholamine–refractory shock support their efficacy in restoring AP and improving tissue perfusion and oxygenation. However, the sparse data on optimal dosing and safety in neonates, as well unknown effects on long-term ND outcomes, do not allow for definite conclusions to be drawn [20].

### 5.6. Levosimendan

Levosimendan, a pyridazinone-dinitrile derivative, is a calcium sensitizer, positive inotropic, and vasodilator agent. It exerts its inotropic action via selective binding to cardiac troponin [92]. Thus, it improves myocardial contractility by enhancing the sensitivity of contractile myofilaments to intracellular calcium in the myocardium [9]. The advantage of LEVO over catecholamines and phosphodiesterase inhibitors is its stimulating effect on cardiac contractility without intracellular calcium overload or an increase in myocardial oxygen demand [92]. Besides its effect on myocardiac contractility, LEVO mediates vascular dilatation through the opening of potassium (KATP) channels in vascular smooth muscle cells, thereby protecting myocardium, liver, and kidney from ischemia–reperfusion injury [92,176]. These properties of LEVO are especially important for patients with severe sepsis and septic shock [176,177]. Other properties of LEVO that protect the myocardium include the modulation of oxidant/antioxidant balance and the protection of mitochondrial function, in addition to direct anti-inflammatory and antiapoptotic cardioprotective properties [36,92]. Moreover, NIRS examination of neonates undergoing cardiac surgery has shown that LEVO improved tissue oxygenation [93].

An important biological characteristic of LEVO is its ability to be metabolized to amino-phenyl-pyridazinone (OR-1855), which is then acetylated to the active metabolite OR-1896. OR-1896 possesses potent inotropic, chronotropic, and vasodilatory properties [94,178,179]. The elimination half-lives of both OR-1855 and OR-1896 (about 75–80 h) are much longer than the LEVO half-life [94]. Consequently, these metabolites augment the hemodynamic effects of LEVO and extend its actions for 7–14 days after cessation of the intravenous infusion [36,180].

Dose regimens used in adults vary widely. A regimen often used in adults comprises a bolus dose of 12.5 mcg/kg over 10 min, followed by infusion of 0.2 mcg/kg/min over 24 h [36]. The estimated dose regimen for neonates, small infants, and children up to 20 kg was a 24 h infusion dose of 0.2 mcg/kg/min [181].

Data regarding LEVO use in neonates and children are limited [36,182,183]. An RCT and an observational study by the same group of researchers showed that in critically ill infants with LCOS, continuous infusion of LEVO increased cerebral perfusion, cerebral and peripheral oxygenation index and decreased serum lactate. In addition, the HR decreased, but the blood pressure did not change [36,182]. The authors also suggested that LEVO may have advantages over MIL in terms of the dosing regimen [36,182]. Ten years later, a large case-series study of preterm infants (n = 105, GA < 37 weeks) with severe cardiac dysfunction and pulmonary hypertension demonstrated that LEVO treatment was associated with a rapid improvement in hemodynamic derangement [184]. Relevant systematic reviews have reported controversial results. They have either shown a beneficial effect of LEVO on cardiac function and early clinical outcomes [185], or no significant difference in hemodynamic effects between LEVO and standard inotrope treatment including DOP, DOB, and MIL [186].

Although data on adverse effects are very limited, clinical studies in neonates/children have reported that LEVO was well tolerated [36]. The observed side effects have included tachycardia, arrythmia, and hypotension; however, the hypotension did not require intervention, or discontinuation of LEVO, only close monitoring [186].

In summary, some published studies showed a potentially beneficial effect of LEVO on cardiovascular function of preterm infants with LCOS and PPHN and its safety. However, current data are controversial, limited, and are of a rather inadequate quality, so that they cannot support any recommendation for the use of LEVO in neonates with severe cardiac dysfunction.

### 5.7. Corticosteroids

Approximately 26 to 48% of hypotensive neonates do not respond to volume resuscitation, vasopressors, and/or inotrope medications, and receive corticosteroids [187]. The decreased, or lack of response to exogenous catecholamine administration, can be partly attributed to downregulation/desensitization of cardiovascular adrenergic receptors, along with relative or absolute adrenal insufficiency in preterm infants [42,43,44,188,189]. In addition, studies in preterm and term infants requiring vasopressor support have demonstrated low cortisol levels (significantly associated with GA), but with a normal response to exogenous ACTH [44,190]. Although the mechanisms underlying the cardiovascular effects of hydrocortisone have not been fully delineated, it seems that both genomic and non-genomic actions are involved. Genomic mechanisms include stimulation or reverse of the desensitization of cardiovascular adrenergic and angiotensin receptors and inhibition of nitric oxide synthase and prostaglandins [188,191,192]. In the frame of non-genomic mechanisms, hydrocortisone induces a rapid increase in AP via improvement in capillary integrity, inhibition of catecholamine metabolism, and increase in intracellular calcium (Table 1) [188,191].

Pharmacokinetic studies, performed in critically ill infants (median GA of 28 weeks) with inotrope–refractory hypotension, showed that the typical half-life for unbound hydrocortisone was 2.9 h [193]. The optimal dose of hydrocortisone for the treatment of neonatal hypotension has not been well-defined. In published studies, different dose regimens have been used: 0.45–0.18 mg/kg/hour or 50 mg/m^2^/day for 2 days followed by weaning over 3–5 days; 100 mg/m^2^/day for 1–2 days, followed by weaning over 4 days [194].

In clinical practice, corticosteroids (hydrocortisone and dexamethasone) are administered as an adjuvant or rescue therapy to neonates with shock not responding to the first- and second-line vasoactive medications [96,98,195,196]. Hydrocortisone is the preferred steroid due to a lower incidence of side effects when compared to dexamethasone. It increases AP within 2–4 h after commencement [96]. The recommended dose of hydrocortisone for preterm infants with refractory hypotension is a loading dose of 1–2 mg/kg, followed by 0.5–1 mg/kg every 8–12 h in preterm infants <35 weeks and every 6–8 h in more mature preterm and term infants (Table 1). The duration of treatment is individualized depending on the cardiovascular response [96,97].

Several RCTs and prospective or retrospective observational studies explored the effects of dexamethasone and hydrocortisone addition as rescue therapy in hypotensive ELBWI and neonates following open heart surgery who did not respond to volume expansion and/or catecholamine (DOP, DOB plus EPI) treatment. Collectively, the results showed that the addition of stress dose of hydrocortisone or dexamethasone efficiently treated refractory hypotension and decreased the duration of inotrope administration [95,96,197,198]. Moreover, no adverse effect on mortality or the incidence of neonatal complications was observed, including severe periventricular–intraventricular hemorrhage (PIVH), periventricular leukomalacia, retinopathy of prematurity, sepsis, NEC, bronchopulmonary dysplasia, gastric bleeding, or intestinal perforation [47,97,98,188,194]. In addition, a retrospective study on term infants with refractory PPHN resistant to inspired nitric oxide (iNO) and inotropes showed that hydrocortisone administration as rescue treatment increased AP, improved oxygenation index and PaO_2_/FiO_2_ (arterial oxygen partial pressure to inspired oxygen fraction) ratio, and decreased the -inotropic score [199].

The most severe short-term side effect of steroids is increased risk of spontaneous intestinal perforation in preterm infants exposed simultaneously to corticosteroids and cyclooxygenase inhibitors given for closing the PDA [96]. Intestinal perforation is more frequent in neonates treated with hydrocortisone [200]. Gastrointestinal bleeding, transient hyperglycemia, hypertension, and the rare cases of myocardial hypertrophy are among the reported short-term adverse effects of corticosteroid administration to preterm infants [96,200]. Data regarding the long-term side effects of corticosteroids are controversial [45,200,201,202,203]. A meta-analysis showed that infants treated with corticosteroids had increased incidence of cerebral palsy, but not combined (death and/or cerebral palsy), except for the subgroup treated with dexamethasone that had a higher incidence of cerebral palsy [204]. Hydrocortisone improved short-term outcomes without adverse long-term ND impairment [204]. Therefore, the potential adverse effects of early corticosteroid treatment on ND outcomes remains a major concern [45,201,203,204].

## 6. Long-Term Outcomes of Hypotensive Neonates Treated with Vasoactive Medications

The limited studies that have assessed the association of neonatal hypotension and related therapeutic and diagnostic interventions during early postnatal life on long-term ND outcomes have reported controversial results (Table 2). Two studies associated early AH (combined with hypoxia in one study) with impaired ND at 12 and 18–22 months of age, respectively, regardless of the treatment given [17,205]. In contrast, another study could not demonstrate any significant association between early AH and adverse long-term ND outcome [18,206]. Three studies associated the inotrope treatment with adverse ND outcomes at 18 months or beyond [13,19,207], while four additional studies did not confirm such an association [12,14,15,59]. Controversial results were also obtained by studies concerning the long-term effects of corticosteroid treatment. Early studies reported that dexamethasone administration to preterm infants was associated with smaller brain volume, which may be linked to impaired ND [96,201]. However, clinical studies in preterm infants receiving early postnatal hydrocortisone for bronchopulmonary dysplasia found that the incidence of adverse ND outcomes at 18–24 months was comparable with that in the placebo group [45,202,203]. Of note, less infants in the hydrocortisone group had a developmental quotient of less than 70 [45,203]. Moreover, a potentially beneficial effect of early hydrocortisone treatment on brain development is supported by the reported association of hydrocortisone treatment with accelerated microstructural organization in the prefrontal and somatosensory cortices. This finding suggests a potentially protective effect of postnatal hydrocortisone on cerebral cortical development in preterm infants [208].

Two additional studies investigating the association of low SVC flow treated with inotropes with long-term outcomes reported controversial results [14,60]. Particularly, a study by Osborn et al. (2007) demonstrated that the combined adverse long-term outcome (death and ND disability) at the age of three years was significantly associated with the low SVC flow during the first few postnatal days, but not with DOP treatment [60]. In contrast, a recent study by Bravo et al. in preterm infants with early findings of low SVC flow treated with either DOP or placebo did not find any significant association between ND impairment at 2 and 6 years of age with either low SVC flow or dopamine treatment (Table 2) [14].

An ongoing multicenter randomized RCT, the SafeBoosC-III clinical trial, aiming to evaluate the association between cerebral oxygenation during the transitional period and ND at two years of life in ELBWI treated for hypotension during the transitional period may provide important information on this issue [210].

## 7. Indications for Anti-Hypotensive Medications in Clinical Practice

The cardiovascular effects of the vasoactive medications in relation to the infant’s cardiovascular status, assessed using functional echocardiography, and the pathophysiology and severity of the underlying neonatal disease, are major determinants of the most appropriate medication for each individual neonate [21,28,32,54,55]. In addition, the degree of immaturity reflecting receptor expression, postnatal age, and the frequency and severity of potential adverse effects should be considered [28].

DOP is the first-line agent for the treatment of hypotension of unknown origin or secondary to perinatal asphyxia or warm septic shock (characterized by peripheral vasodilation) [106,195,211]. Additionally, due to the vasoconstrictive effect of DOP on both the systemic and pulmonary circulation, it is indicated for the treatment of hypotension in neonates with a large PDA not responding to pharmacological closure [58,113,212].

DOB is used in 20 to 33% of VLBWI, usually as a second-line treatment, an adjuvant to DOP, or as rescue treatment when DOP fails to increase AP at an infusion rate up to 15 mcg/kg/min [49,58,102]. Due to its inotropic action on myocardium, DOB is the medication of choice in cases with cardiac dysfunction. It is suggested as an appropriate medication for the treatment of hypotensive preterm infants during the transitional period [106]. Moreover, DOB’s effectiveness in decreasing pulmonary vascular resistance and increasing myocardial contractility makes it a potential first-line medication in neonates with PPHN associated with myocardial dysfunction, to restore cardiac output and reverse the associated myocardial dysfunction [30,58,62,195,213]. The low systemic blood flow associated or not with a hemodynamically significant PDA is an additional indication for DOB administration [16,60].

EPI is usually administered as a third-line medication in neonates with hypotension not responding to the first- and second-line medications, e.g., DOP and DOB [214]. However, in clinical practice, it has also been used as a first-line anti-hypotensive treatment [9]. Low dose EPI (0.02–0.1 mcg/kg/min) is recommended for the treatment of cold septic shock due to its properties to increase myocardial contractility and dilate peripheral vascular beds [40]. In warm septic shock, which is characterized by systemic vascular dilation and consequent decrease in vascular resistance, high doses of EPI (>0.1 mcg/kg/min) are indicated to cause systemic vasoconstriction leading to increased systemic vascular resistance [66]. An RCT in neonates with warm septic shock showed that EPI was comparable to DOP in increasing AP, preserving hemodynamic stability, and improving metabolic acidosis [211].

The main indication of NE includes its use as rescue treatment of severe shock, predominantly septic shock refractory to other catecholamines (DOP, DOB, and EPI) [54]. It is suggested that NE should be added quickly after failure of dopamine. In addition, studies in adult patients with septic shock showed that NE more effectively reversed hypotension and it was associated with lower mortality and lower incidence of adverse effects, compared to other inotropes [195,215,216,217]. Therefore, the Surviving Sepsis Guideline panel, a consensus committee of 55 international experts representing 25 international organizations, recommended NE as the first-line inotrope in severe septic shock, cardiogenic shock, or non-identified shock [133]. The time of NE initiation also affects the outcome, as it was reported that early initiation of NE in patients with septic shock was associated with decreased short-term mortality and shorter time to achieved target MAP [218,219]. Based on these data, studies in neonates suggest NE as a first-line agent in septic shock [54,68,70,71,132], which should be implemented quickly to prevent the deterioration of cardiovascular function [218,220]. In this context, in neonates with sepsis, early recognition of the first clinical signs indicating a potential progress to septic shock, such as deviation of AP from basal values even when it remains higher than the limit of normal range, is of utmost importance [221]. Combining NE with other vasoactive medications improves the outcome of patients with septic shock [222]. Severe PPHN with consequent cardiovascular failure acts as another indication for NE use. Relevant studies in neonates with PPHN showed that administration of NE decreased both the pulmonary and systemic vascular resistance, leading to improved pulmonary blood flow and oxygenation [132].

MIL is also a medication of choice for patients with low cardiac output syndrome (LCOS) and increased peripheral resistance, as well as neonates with PPHN, congenital diaphragmatic hernia, and low systemic blood flow complicating PDA ligation [24,93,162]. In such patients, treatment with MIL improves cardiac function and reduces pulmonary vascular resistance and oxygen requirement. However, studies in adults showed that MIL may cause hypotension [30,223]. The risk of hypotension is increased in the presence of certain neonatal morbidities, including very low GA, organ dysfunction, and perinatal asphyxia [30]. Therefore, it has been suggested that MIL must be administered cautiously, potentially combined with other inotropes to retain hemodynamic stability [73,74,142,147].

The main indication for AVP is its use as a single agent, or an adjuvant treatment, for catecholamine refractory shock, predominantly in neonates with low systemic vascular resistance, e.g., neonates with warm septic shock [195]. In addition, its potent vasoconstrictive properties on systematic circulation and selective pulmonary vasodilatory effect make AVP the medication of choice as a first-line agent for neonates with either PPHN without cardiac dysfunction, or low systemic blood flow secondary to sepsis and post-surgery for open correction of congenital heart disease [171].

The DOP- and DOB-refractory septic shock is the main indication for hydrocortisone administration as an adjuvant or rescue therapy, due to its beneficial effects on receptor immaturity and capillary leak [96]. In a study by Ng et al. (2006) on 48 VLBWI with hypotension non-responsive to DOP at a dose of 10 mcg/kg/min, a stress dose of hydrocortisone decreased the duration of vasopressor support [98].

Giesinger et al., in an excellent review, suggested that a separate assessment of systolic and diastolic AP, instead of only the mean AP, may be of valuable assistance in deciding which medication is the most appropriate for each neonate [30]. PPHN, sepsis, and cardiogenic shock are characterized by low left ventricular output, resulting in systolic hypotension. In such cases, vasoactive drugs with positive inotrope action on the myocardium (e.g., DOP at medium doses) are the preferred medications. The presence of low diastolic AP indicates low vascular resistance, such as in hypovolemia, sepsis, or PDA. Medications causing peripheral vascular constriction should be administered, including AVP, EPI, NE, and DOP [28,30]. Finally, if both systolic and diastolic AP are low, the pathophysiology is more complex, involving low left ventricular volume and systemic vascular resistance, while the systolic function of the heart may be disrupted [28,30]. Neonatal diseases characterized by low systolic and diastolic AP include PPHN, PDA, cardiogenic shock, previously low systolic or diastolic AP, hypovolemia, and warm shock [28,30]. In such cases, medications decreasing the pulmonary vascular resistance and/or increasing systemic vascular resistance, such as MIL, EPI, and NE, are the most appropriate anti-hypotensive drugs [28]. In fact, a retrospective study in 1446 infants exposed to MIL during a 14-year period found that the most frequent neonatal situation needing MIL treatment was the PPHN (40%) [77]. Neonates with PPHN resistant to these inotropes may benefit from AVP administration.

In the context of complexity of neonatal hypotension pathophysiology, the choice of the most appropriate management is still a matter of debate requiring further in-depth understanding of the underlying mechanisms. Therefore, certain groups of researchers provided algorithms for the assessment and treatment of hypotension in neonates with various morbidities [28,30,106].

Recent studies have examined the contribution of artificial intelligence and machine learning techniques in decision making for the most appropriate treatment approach, and for predicting the effectiveness of different vasoactive medications for each individual patient. These techniques consider the patients’ characteristics and potentially known responses to vasoactive drugs, the pathophysiology underlying the development of AH, as well as laboratory, monitoring, and environmental data [224,225,226]. In fact, very recently, Bravo et al. (2023) presented a prediction model created using machine learning techniques [226]. The proposed model could correctly predict the effectiveness of DOB in 90% of the responding VLBWI, while the effectiveness DOP was anticipated in 61% of cases [226]. Additional relevant studies performed by cooperating scientists of different specialties, such as neonatologists, biomedical informatics specialists, and epidemiologists, have been recently published [224,225,226].

## 8. Comparisons between Anti-Hypotensive Medications

During the past decades, several clinical studies and systematic reviews compared the effectiveness and safety between the anti-hypotensive medications used in neonates to facilitate the choice of the most appropriate anti-hypotensive medication (Table 3). More details are provided in the Supplementary Table S1.

*Dopamine vs. placebo or plasma protein fraction*. Two early studies comparing DOP with placebo (dextrose in water) or volume expansion (using plasma protein fraction) in 14 severely asphyxiated term neonates and 39 hypotensive VLBWI, respectively, showed that DOP was more effective in increasing AP and improving echocardiographic indices without any significant change in HR or systolic time intervals [209,227]. These findings were confirmed by systematic reviews [61,232].

*Dopamine vs. dobutamine*. Several RCTs, case-control studies, case series, and systematic reviews, mostly referring to early AH treatment of preterm neonates, compared the effectiveness and safety between DOP and DOB. It was found that treatment with DOP increased mean AP in a dose-dependent manner, more efficiently than DOB, while DOB was more efficient in increasing the low SVC flow [56,58,228,233]. No significant difference in mortality and morbidity [58], nor on ND outcome at 1 and 3 years of age [60], was found. Moreover, DOP, but not DOB, was associated with suppression of TSH, T4, and prolactin that was reversed soon after treatment stopped [116]. These findings were confirmed by systematic reviews and meta-analyses that regarded existing data insufficient to support a definite suggestion, mainly due to sparse data on the long-term ND effects [16,61].

*Dopamine vs. epinephrine*. Three RCTs conducted by the same research group in hypotensive preterm neonates (GA < 32 weeks) that received early treatment with DOP or EPI found that both medications were equally effective in increasing the AP and improving left ventricular output, cerebral hemodynamics, and clinical outcomes [59,65,66]. Moreover, a comparable response rate and need for rescue therapy were demonstrated. However, the EPI group presented significantly higher HR, plasma lactate, base deficit, blood glucose, and needs for insulin treatment. No difference in the medium-term morbidities and ND outcomes at the age of 2 to 3 years was reported [59,65,66]. A more recent randomized clinical trial (RCT) explored the effects of EPI versus DOP as a first-line inotrope treatment in 40 neonates with volume-refractory septic shock [211]. There was no significant difference between the two treatment groups regarding the frequency of shock reversal, AP, hemodynamic stability, duration of inotropes, prevalence of complications of prematurity, and all-cause mortality [211]. Three systematic reviews and meta-analyses confirmed the results of RCTs and concluded that there was no evidence supporting the superiority of either inotrope [230,232,234].

*Dopamine vs. norepinephrine*. Only one published, retrospective study compared the clinical effects of DOP versus NE as first-line therapy in 156 very low GA infants with septic shock [69]. It was found that treatment with NE was related to lower mortality, as well as incidence of significant neurologic injury and sepsis/NEC among survivors [69].

*Dopamine vs. vasopressin*. Rios et al. compared the effectiveness in increasing AP between DOP and AVP as initial therapies in hypotensive ELBWI during the first 24 h after birth. The response rate (90% for both treatment groups) and response time were comparable between the two groups. However, the AVP group did not develop tachycardia during infusion, required fewer doses of surfactant, and had a lower PaCO2 compared to DOP group [78].

*Dopamine vs. hydrocortisone*. An early study in 40 hypotensive VLBWI showed that administration of either hydrocortisone or DOP as a first-line therapy resulted in a comparable response rate (100% and 81%, for DOP and hydrocortisone, respectively) without any difference in neonatal morbidities (PDA and sepsis), except for hyperglycemia that was more frequent in the hydrocortisone group [229]. More recent studies in VLBWI and term neonates with various morbidities (septic shock, perinatal asphyxia, meconium aspiration syndrome) presenting with DOP-, DOB-, and EPI-refractory hypotension showed that the addition of hydrocortisone resulted in a greater increase in AP, reduced mortality, and reduced duration and dosage of cardiovascular support compared to placebo. No increase in the incidence of neonatal complications was observed in the hydrocortisone group, including severe PIVH or periventricular leukomalacia [97,98].

*Dobutamine vs. placebo*. An exploratory trial of 127 preterm neonates (GA < 31 weeks) with low SVC flow observed using serial echocardiography, showed that compared to placebo, DOB infusion increased AVC flow and HR, and decreased base excess. There was no difference in the incidence of the composite outcome (mortality and ND) at six years of life [14,113].

*Dobutamine vs. milrinone*. There is no study in neonates comparing DOB with MIL. Instead, an RCT pilot study in 50 children (age 2.5 months to 14.2 years) undergoing cardiac surgery reported that both medications were equally effective in preventing LCOS and both were well tolerated [149].

*Epinephrine vs. no treatment*. An early Cochrane review that compared the effectiveness and safety of EPI versus no treatment, or other inotropes, in preterm neonates with cardiovascular compromise did not find any relevant studies [230].

*Epinephrine vs. hydrocortisone*. A recent multicenter study in neonates with septic shock receiving DOP reported that mortality rate increased significantly following the addition of EPI, but decreased when hydrocortisone was added [47].

*Milrinone vs. placebo or no treatment*. An RCT comparing the effect of MIL versus placebo in preventing LCOS in high-risk neonates and children (aged 2 days to 6.9 years) after corrective cardiac surgery showed that MIL significantly reduced the risk of LCOS [140]. In contrast, a subsequent RCT and a retrospective study did not demonstrate any beneficial effect of MIL on hemodynamic markers, morbidity, or mortality compared to placebo [145], or no prophylaxis with MIL [136].

*Milrinone vs. levosimendan*. Most RCTs comparing the pharmacodynamic effects of MIL versus LEVO could not find any significant difference concerning the hemodynamic effects between the two inotropes [36,150,235]. Only the RCT by Momeni et al. (2011) involving 36 children (0–5 years old) operated on for congenital heart disease, showed that the LEVO group had significantly lower myocardial oxygen demands, and a trend towards lower troponin levels postoperatively [183]. LEVO was well tolerated and was associated with a lower incidence of side effects and certain short-term clinical outcomes [36,150,183,235]. Interestingly, LEVO had sustained hemodynamic effects after the drug withdrawal, attributed to its long-lasting active metabolites, which could be detected in plasma up to day 14 post-surgery. The authors concluded that LEVO may have advantages over MIL in terms of the dosing regimen [36].

*Levosimendan vs. placebo*. A recent RCT examining the effectiveness of LEVO in the prevention of LCOS in 94 infants older than 1 month (2 to 16 months) undergoing cardiac surgery versus placebo (n = 93) did not show any significant benefit of LEVO with respect to the prevention of LCOS, 90-day mortality, or other clinical outcomes [236].

*Levosimendan vs. standard inotrope treatment*. An RCT compared the effect of LEVO versus standard inotrope treatment (MIL and DOB, with or without EPI) on preventing LCOS in newborns during the post-operative period of heart surgery. It was reported that the LEVO group had lower incidence of LCOS and postoperative HR, lactate levels, and inotropic score. No significant difference in mortality, length of mechanical ventilation, or length of stay in pediatric cardiac intensive care unit was observed. LEVO was well tolerated [93]. However, a Cochrane systematic review of five RCTs with a total of 212 participants under 5 years (0 to 18 years) could not find any significant difference between LEVO and standard treatments regarding the prevention of LCOS and mortality [186].

*Hydrocortisone vs. placebo*. Two RCTs and one prospective cohort study in preterm infants with hypotension refractory to volume and inotropes (DOP and/or DOB plus EPI), compared the effects of the addition of dexamethasone [231] or hydrocortisone [98,194] versus placebo. It was found that the addition of dexamethasone or hydrocortisone was associated with an improvement in cardiovascular status and a significant decrease in the duration of inotrope support [98,194,231]. No study reported any side effects of corticosteroids on mortality and neonatal morbidity [98,194,231]. Likewise, a placebo-controlled RCT in 35 term neonates with perinatal asphyxia receiving hypothermia who developed DOB-refractory hypotension showed that the addition of hydrocortisone resulted in a greater increase in AP and a reduced duration of cardiovascular support, as well as decrease in cumulative and peak inotrope dosage, compared to placebo [97].

## 9. Discussion

The cardiovascular support of neonates has constituted a constant challenge throughout the past four decades [11,237]. Most studies involve very premature neonates with early cardiovascular insufficiency apparently due to structural and developmental aspects of the cardiovascular system and the transitional changes that occur over the first weeks of life [61]. However, RCTs performed to investigate the role of cardiovascular medications in other neonatal conditions are alarmingly sparse. Multicenter studies have demonstrated a high variation in inotrope use among different NICUs, and it is inversely proportional to GA: 93% and 73% of preterm infants with GA 23 weeks and 27 weeks, respectively [4,10,11,237,238]. This variation indicates a lack of established management of AH in neonates, which can be partly attributed to the varying published reference ranges of AP and definitions of AH.

Previous studies reported certain drawbacks of methodologies used in different studies that have contributed to inconsistencies in reference ranges of AP, especially when data were collected retrospectively. Methodological differences affecting interpretation of the reported AP in neonates include, but are not limited to, the retrospective design and differences in the participant populations (such as small samples in different GA subgroups, grouping together a wide range of GA, different postnatal ages, inclusion of growth restricted newborns, and clinical condition). In addition, the inclusion of infants treated with inotropes/vasopressors, differences in reporting of AP (i.e., systolic and diastolic versus mean), lack of longitudinal assessment, difference of measurements (frequency/timing), and the exclusion of neonates with poor outcomes contribute to the inconsistencies between studies. Moreover, AP values may differ depending on the method of measurement [7,9].

The important differences and limitations of the published study design, along with the complex pathophysiology of AH in preterm infants, add to the difficulties in defining and treating AH in this fragile population [7,20,26]. In fact, a prospective observational study showed that 3–49% of ELBWI with low AP did not receive anti-hypotensive treatment; however, 28–41% of infants without low AP were treated. These data indicate that management approach was not based only on AP values, but that additional clinical and biochemical parameters, and potentially echocardiographic and NIRS findings, were also considered [239]. Ensuring perfusion and oxygenation of major organs, mainly the heart, brain, and kidneys, is a major target of AH treatment [2,20,27]. Published data suggest that AP alone cannot accurately indicate cerebral perfusion and the need for cardiovascular support in VLBWI [27]. A recent prospective randomized controlled trial (HIP trial) in ELBWI with AH showed that treatment with DOP increased the mean AP but did not improve cerebral oxygenation. Moreover, it was found that the duration of cerebral hypoxia, secondary to cerebral hypoperfusion, was significantly associated with PIVH or death [240]. These findings partly confirmed preceding results of a Cochrane systematic review, which included five studies with 4754 ELBWI. The authors concluded that targeting lower (85% to 89%) versus higher (91% to 95%) oxygen saturation had no significant effect on long-term ND outcome, while targeting low oxygen saturation was associated with increased mortality rate by 28 per 1000 infants treated [26]. A following systematic review by the same research group demonstrated that abnormal cerebral NIRS did not correlate with either the hypotension or the PIVH grades, mortality, average hematocrit, serum lactate, and urine output [241]. Although relevant data for other vasoactive medications are lacking, several authors suggested that monitoring of organ perfusion and assessment of cardiovascular hemodynamics could potentially guide the neonatologist not only for the need of inotrope/vasopressor administration, but also for an individualized, pathophysiology-based choice of the most appropriate anti-hypotensive medication [20]. It should be noted though that whether close monitoring and keeping cerebral perfusion/oxygenation within the target range improve short- or long-term outcomes is still debated [20,27,212,242,243]. A currently underway RCT in preterm neonates (<32 week GA), the COSGOD phase III trial, may help clarify the effect of targeting cerebral oxygen saturation during the transition period on mortality and/or cerebral injury [244]. Until then, it is recommended that a physiology-based definition of AH should consider both cerebral perfusion and cardiovascular function using targeted echocardiography and NIRS monitoring, alongside AP values and clinical and laboratory findings [26,28,245].

The literature regarding the anti-hypotensive treatment of neonates includes mainly retrospective studies, either cohort or case-control, and review articles. Available RCTs are limited and include relatively low numbers of neonates, due to obstacles frequently encountered when attempting to perform research in neonates. A major problem reported is the difficulty in obtaining informed parental consent before or soon after birth, with only 17% to 30% of eligible neonates being enrolled and studied. In addition, participating clinicians were rather unwilling to recruit preterm neonates, considering them too ill for participation in the study [239,246]. These difficulties were leading causes of the early ending of at least two multicenter randomized trials: the HIPHOP trial in Italy and the NICHD hypotension trial in USA [246,247,248].

In the context of the reported obstacles in performing research in neonates, major questions regarding when, why, and which anti-hypotensive medications to use remain. The first question concerns the need for a generally accepted definition of AH during and beyond the transition period, in relation to updated AP reference values, cerebral perfusion, and the potentially co-existing neonatal morbidities.

Another important challenge is related to whether the low values of AP during the transition period constitute a real health problem needing treatment, or if they simply reflect the physiologic adaptation of the neonatal circulation from the intrauterine to the extrauterine environment. It has been shown that the use of vasoactive medications is higher during the transition period, despite the existing controversy as to the need of anti-hypotensive treatment and the potential adverse effects of AH and/or anti-hypotensive treatment on short- and long-term outcomes [12,41,49,247]. In the context of the existing controversies, some neonatologists suggested an abstinence from intervention for isolated hypotension during the first 3 days of life in ELBWI with concomitant cautious assessment of clinical condition and close monitoring of cardiovascular status and cerebral perfusion [41]. The limited and inconsistent data on the association of low AP and vasoactive medication use during the transition period with ND outcome cannot support any recommendation regarding a cautious management approach of low AP in this period of life [41,60,205]. The COSGOD III trial that is currently underway may provide additional insight on this issue [244].

Another problem needing special consideration when treating hypotensive neonates is the reported disproportion between dose administered and plasma concentrations of certain inotropes, such as DOP and DOB, as well as the wide inter-individual variation in plasma concentrations and clinical response following infusion of a similar dosing regimen [64,103]. For this reason, it has been suggested that infants treated with inotropes should be closely monitored to adapt the dosage to the cardiovascular response. In fact, a stepwise increasing infusion rate was associated with increases in plasma levels of DOB, while echocardiography showed a parallel improvement in cardiac systolic function and hemodynamic variables [106]. However, the wide variation observed in hemodynamic and pharmacokinetic data highlights the need for individualized inotropic dosing [115].

Among the interventions for cardiovascular support used in NICUs, volume expansion is the most common first-line intervention. The role of this procedure may be overestimated since hypovolemia is not a frequent problem in preterm infants within the first week of life. Volume expansion is indicated only in rare cases of preterm infants with perinatal blood loss [32]. On the other hand, fluid infusion of 10–20 mL/kg within 30–60 min can increase preload, and consequently the cardiac output in preterm infants, thereby decreasing the need for vasoactive medications, regardless of the presence of hypovolemia [32,49]. Hypovolemia may also be a problem in neonates with sepsis due to increased capillary leak.

Treatment with inotropes and vasopressors is a long-established intervention in neonates with AH not responding to volume expansion. However, an important question concerns the existing controversy surrounding the potential association of AH and inotrope treatment with long-term ND outcomes. Thus far, it has not been fully understood whether the observation in some studies of adverse long-term outcomes should be attributed to the low AP and the associated organ hypoperfusion/hypoxia, the anti-hypotensive treatment itself, the pathophysiology of underlying disease process, or a combination of several known and unknown factors. As a result, the vast majority of the relative clinical studies and systematic reviews conclude that existing data cannot support or refute the use of certain anti-hypotensive medications and suggest the design of additional larger clinical trials with high statistical strength [16,30,40,61,63,83,92,102,171,241,249].

Due to insufficient available data regarding safety and the lack of age-appropriate doses and formulations for neonates, the vast majority of currently utilized anti-hypotensive medications are not licensed for use in neonates [250,251]. Nevertheless, vasoactive medications are used off-label in NICUs worldwide [28,214,252]. The off-label use is supported by a European Commission regulation that encourages the off-label use in the pediatric population for research purposes [251,253].

An additional important challenge lies in the potential genetic predisposition underlying the development of AH, which may explain differences in the response of individual patients to anti-hypotensive medications. Interestingly, polymorphisms of certain genes associated with adrenergic and other receptor functions, as well as epigenetic DNA modifications, have been found in infants treated for early hypotension [191,254]. Specifically, Hallik et al. revealed *beta-1-AR Arg389Gly* and *GNAS c.393C > T* polymorphisms in DOB-treated infants, while Kantake et al. showed that corticosteroid treatment was the most significant independent factor positively associated with changes in methylation rate [191,254].

## 10. Future Perspectives

In the context of reported disagreement between AP and cerebral perfusion, extended use of targeted neonatal echocardiography and cerebral NIRS to evaluate the cardiovascular integrity and cerebral perfusion may considerably improve the assessment of neonatal circulation and indicate an individualized, age- and physiology-based anti-hypotensive treatment strategy [51]. Currently, ongoing studies are expected to improve our understanding of this issue [210,244]. For the time being, the lack of sufficient number and size of well-designed RCTs in neonates, combined with the observed differences in the study design, definitions, participating populations, and measurement methods prevent the composite analysis of published studies and comparisons between vasoactive drugs by systematic reviews. Moreover, studies on the long-term effects of early neonatal hypotension and vasoactive medications are sparse. Therefore, all systematic reviews have reached the same conclusion stating that existing data cannot support or refute the use of the anti-hypotensive medications to neonates. Nevertheless, thus far, certain groups of researchers have developed flow charts with guidelines to help with the evaluation and treatment of neonatal hypotension in clinical practice [28,30,106]. The generalized acceptance of the guidelines developed by international neonatal/perinatal scientific societies and working groups of experts can guide neonatologists regarding the appropriate management of hypotension in clinical practice [22,137]. Moreover, the provision of a well-designed unified study protocol and creation of a universally available database are required to improve the conduction of multicenter studies with comparative results. The use of artificial intelligence and machine learning models, such as those created recently [210,244], may dramatically change the way hypotension is treated, and will contribute to the establishment of evidence-based guidelines for a best-management approach of neonatal cardiovascular instability in clinical practice.

## 11. Conclusions

The complex and multifactorial pathophysiology of circulatory instability, the reported disproportionate correlation of certain vasoactive medications dosage with a hemodynamic effect on cardiovascular status in neonates, and the varying thresholds of hemodynamic factors that may potentially cause irreversible long-term ND impairment outline the need for a personalized management approach. To this aim, besides the AP, the assessment of clinical condition, laboratory testing, and specialized monitoring have been included in the evaluation of cardiovascular function in neonates [28,255]. The addition of artificial intelligence and machine learning models to the existing diagnostic tools is expected to improve the diagnosis of cardiovascular derangement [210,244], and contribute to the establishment of evidence- and pathophysiology-based guidelines for a best management approach of neonatal cardiovascular instability.

## Figures and Tables

**Table 1:** Characteristics and clinical data of anti-hypotensive medications used in neonates.

Medication [References]	Mechanisms of Action/Receptors	Hemodynamic Effects	Dose Regimen (Range) *	Main Indications	Potential Side Effects
Dopamine [9,40,51,56,57,58,59,60]	D1R/D2R	VD of peripheral vascular beds. Increases GFR and tubular functions; endocrine effects; induces EPI and NE production.	≤2–5 (2–20)	Most common first-line agent for systemic AH in the absence of cardiac dysfunction.	Tachycardia: at doses ≥10, may cause PPHN; decreased production of TSH, GH, and prolactin.
	DR > β-1R > α-1R	Positive inotropic and chronotropic; increase HR, AP, myocardiac contractility, and CBF.	≥5–10 (2–20)		
	α-1R > β-1R	VC; increase AP and PVS and SVR.	≥10–20 (2–20)		
Dobutamine [9,14,49,51,56,61,62,63,64]	β-1R > β-2R > αR	Inotropic effects and VD; increase cardiac contractility and output. Decrease PVR and SVR.	2.5–5 (2.5–10)	First- or second-line agent or combined with DOP; AH with cardiac dysfunction; transitional AH with low cardiac output and increased SVR or large PDA; PPHN with right ventricular dysfunction.	Tachycardia
Epinephrine [9,40,50,65,66,67]	β-1R > β-2R	Inotropic action: increase cardiac contractility and SVR.	0.01 to 0.1 (0.05–2.5)	Adjuvant or rescue treatment for inotrope—refractory shock; PPHN with systemic AH; warm septic shock with decreased contractility; transitional AH with large PDA; rarely, first-line agent.	High doses (over 0.5): increased plasma glucose & lactate
	α-1R > β-1R = β-2R	VC. Increase myocardiac contractility, SVR, HR, AP.	0.1 to 0.2 (0.05–2.5)		
Norepinephrine [51,53,68,69,70,71,72]	α-1R and α-2R	Vasopressor effects; inotropic action and systemic VC. Increases AP and decreases inotrope score. Mild pulmonary VD.	0.01–0.04 (0.04–1)	Adjuvant in catecholamine refractory shock: warm septic shock, PPHN, post-cardiac surgery, perinatal asphyxia; AH with cardiac dysfunction; Maybe 1st choice for septic shock.	VC; transient systemic hypertension; potential tissue necrosis if extravasated.
Milrinone [51,73,74,75,76,77]	Phosphodiesterase inhibitor: increases intracellular cyclic AMP and calcium.	Inotropic and lusitropic actions; VD; decrease in pulmonary and systemic vascular resistance.	LD: 50; Infusion: 0.75 for 3 h. (0.25–0.75)	PPHN post-cardiac surgery; prevention of low LVOS after PDA ligation.	Tachycardia and hypotension requiring vasopressors.
Vasopressin (AVP) [51,78,79,80,81,82]	Antidiuretic hormone. V1R: systemic VD. V2R: selective VD of pulmonary, cerebral, and coronal vascular bed.	Increase AP in catecholamine-resistant shock and cardiac output. Potentially decrease pulmonary vascular resistance.	0.00001 and 0.003 units/kg/min and 0.003 U/kg/min. (0.00001 and 0.003 U/kg/min)	Catecholamine- and steroid-refractory or VD shock, warm septic shock, PPHN, post-cardiac surgery.	Tachy-arrhythmias, hyponatremia; transient thrombocytopenia; liver necrosis. High doses: reductions in cardiac output and oxygen delivery.
Terlipressin [80,83,84,85,86,87,88,89,90,91]	Comparable to AVP. Higher affinity to V1R, longer half-life	Comparable to AVP	LD: 5–20 MD: 5–20 every 4 h	Volume and catecholamine–refractory shock.	Potentially comparable to AVP. Potential limb necrosis. Well-tolerated.
Levosimendan [36,92,93,94]	Calcium sensitizer; inotropic action via binding to cardiac troponin; VD action.	Increase myocardial contractility, AP, tissue perfusion and oxygenation; VD; decrease lactate and HR.	Infusion of 0.2 over 24 h.	Severe, refractory warm septic shock; LCOS; severe cardiac dysfunction and PPHN.	Tachycardia, arrythmia, hypotension potentially requiring intervention. Well tolerated.
Hydrocortisone [39,45,95,96,97,98]	Hormone; stimulation or reverse pf desensitization of AR; improvement in capillary integrity.	Increases AP, improves left ventricular function and oxygenation index, and decreases the need for vasopressors.	Initial: 1–2 mg/kg; MD: 0.5–1 mg/kg every 8–12 h	Adjuvant or rescue therapy for refractory AH (e.g., septic shock, perinatal asphyxia). PPHN resistant to iNO and inotropes.	Transient hyperglycemia, hypertension, spontaneous intestinal perforation, rare myocardial hypertrophy.

* Doses are expressed as mcg/kg/min unless otherwise stated. AH, arterial hypotension; AP, arterial pressure; AR, adrenergic receptors; CBF, cerebral blood flow; D1R/D2R, dopaminergic receptors 1/2; h, hour/s; GH, growth hormone; HR, heart rate; LCOS, low cardiac output syndrome; LD, loading dose; LVOS, left ventricular output syndrome; MD, maintenance dose, PDA, patent ductus arteriosus; PPHN, persistent pulmonary hypertension of the neonate; PVR, pulmonary vascular resistance; SVR, systemic vascular resistance; TSH, thyroid-stimulating hormone; V1R, vasopressin receptor 1; V2R, vasopressin receptor 2; VC, vasoconstriction; VD, vasodilation; αR, alpha adrenergic receptors; α-1R, alpha-1 adrenergic receptors; α-2R, alpha-2 adrenergic receptors; β-1R, beta-1 adrenergic receptors; β-2R, beta-2 adrenergic receptors.

**Table 2:** Studies on long-term neurodevelopmental (ND) outcomes in relation to hypotension, anti-hypotensive treatment, and low superior vena cava flow.

First Author (Year) [Reference]	Design/Aim	Population	Age at Exposure/Age at FU	Main Results	Authors’ Conclusions/Comments
DiSessa et al. (1981) [209]	RCT/AIM: Cardiovascular effects of DOP vs. PL in asphyxiated neonates.	14 asphyxiated term neonates	Early postnatally/1–24 mo	DOP vs. PL: higher increase in AP, improved caECHO indices; 5/6 infants had a normal ND on FU.	DOP raises systemic AP and improves cardiac function.
Watkins et al. (1989) [206]	Cohort/AIM: Assoc. of AH periods with PIVH and ischemic lesions.	131 VLBWI (GA 24–34 weeks); 56 (58% hypotensive)	First 4 DoL/2 years of age.	No association of AH with subsequent NDI in survivors.	Ischemic lesions did not correlate with periods of hypotension.
Low et al. (1993) [17]	Cohort/AIM: AH/hypoxemia assoc. with ND at 1 year.	93 neonates (GA < 34 weeks), 2 Gr: 49 normal ND; 49 abnormal ND.	First 4 DoL/1 year	Major NDI: 8% without AH or hypoxemia vs. 53% with both AH and hypoxemia.	Combination of hypotension and hypoxemia are associated with long-term NDI.
Fanaroff et al. (2006) [207]	Retrospective/AIM: Assoc. of treated AH with ND at 20 mo CA.	156 ELBWI (birth weight 400–999 g).	First 72 hoL/20 mo CA	Early outcomes: Treated AH assoc. with severe PIVH, longer hospital stay, and death. 20 mo CA outcome: Treated AH was assoc. with MDI < 70 and hearing loss.	Infants with treated hypotension are more likely to have delayed motor development, hearing loss, and death.
Osborn et al. (2007) [60]	RCT/AIM: Assoc. of early SVC flow (caECHO) and DOP vs. DOB with ND at 1 and 3 years.	84 neonates (GA < 30 weeks) in 2 Grs: 84 normal SVC flow Gr vs. 44 low SVC flow Gr.: DOP-Gr vs. DOB-Gr.	24 hoL/1 & 3 years.	(A) DOB vs. DOP: sign. lower rate of severe PIVH & disability at 3 year. No sign. dif. in clinical outcomes. (B) Low vs. normal SVC flow: increased mortality, morbidity, severe PIVH, and NDI at 3 year CA.	(A) No sign. dif. in combined death and disability between DOP & DOB. (B) Early low SVC flow was assoc. with increased rates of death, morbidity, and NDI.
Batton et al. (2009) [205]	Retrospective cohort/AIM: Assoc. of early AP treatment with NDI.	168 ELBW (GA 23–25 weeks) in 3 Grs	First 72 h of life/18 to 22 mo PMA	Compared to untreated-normotensive, the untreated-low AP had more CP, deafness, or any NDI and the treated-low AP had worse NDI and less survival without NDI.	Early low AP alone is associated with adverse long-term ND outcomes regardless of treatment.
Kuint et al. (2009) [19]	Retrospective, case control/AIM: Identify risk factors for AH and relations to short- and long-term outcomes.	218 survived VLBWI: TGr (n = 109) vs. CGr (normotensive, n = 109).	24 h of life/ > 2 year	TGr vs. CGr: PIVH 2–4/PVL 11% vs. 2.7%; major disability: 20% vs. 5%	Early anti-hypotensive therapy is related to PIVH, PVL, and major NDI.
Pellicer et al. (2009) [59]	RCT (3 Grs). AIM: Effect of early treatment with DOP or EPI vs. no treatment on ND.	60 hypotensive VLBWI (GA < 32 weeks) and 70 normotensive (CGr).	First 96 hoL/2–3 years of age.	DOP vs. EPI: No differences in NDI (death or CP or severe NDI).	Cautious use of cardiovascular support to treat early AH in LBWI seems to be safe.
Logan et al. (2011) [18]	Prospective cohort/AIM: Assoc. of AH and treatment with ND.	945 neonates (GA < 28 weeks).	First 24 hoL/24 mo CA.	Adjusted AH was not associated with MDI < 70 or PDI < 70.	There is little evidence that early AH is assoc. with NDI at 24 mo CA.
Alderliesten et al. (2014) [12]	Prospective, observational case-control/AIM: Relation of early DOP and cerebral oxygenation with NDI.	132 neonates (GA < 32 weeks, TGr): 66 hypotensive (volume vs. DOP); and CGr, 66 normotensive.	First 72 hoL/18 and 24 mo CA.	NDI was comparable between TGr & CGr but was assoc. with duration of low cerebral oxygenation.	Early hypotension was not assoc. with lower cerebral oxygenation or NDI.
Batton et al. (2016) [13]	Prospective observational/AIM: Relation of early AP and its therapy, with ND.	158 survivors ELBWI (GA 23–26 weeks) in 4 groups.	First 24 h of life/18–22 mo CA.	Death or NDI was sign. higher in the treated as compared to untreated infants irrespective of AP changes.	Exposure to anti-hypotensive therapy was assoc. with increased risk of combined death and NDI at 18–22 months’ CA.
Bravo et al. (2021) [14]	RCT/AIM: Assoc. of early low SVC flow within with long-term outcome.	28 neonates (GA < 31 weeks) with (a) low SVC flow; (b) 98 with normal SVC flow.	First 24 hoL/2 and 6 years.	No sign. dif. in combined outcome (mortality or NDI) between the DOB, PL, and normal flow groups.	No dif. in long-term outcome related to SVC or its treatment early after birth. SVC flow was not associated with long-term NDI
Doucette et al. (2022) [15]	Retrospective cohort/AIM: Examine ND relation with inotrope treatment.	1394 survivors (GA < 29 weeks): TGr, 245 treated with inotropes.	First week of life/18–24 mo of age	Compared to CGr, the TGr had higher rates of hearing loss. No difference in the risk of adjusted NDI.	Inotrope treatment in the first WoL were at increased risk for hearing loss. There was no diff. in NDI.

AH, arterial hypotension; AP, arterial pressure; assoc., associated/ion; CA, corrected age; caECHO, echocardiography; CGr, control group; CP, cerebral palsy; DoL, day of life; DOP, dopamine; DOB, dobutamine; ELBWI, extremely low birth weight infant(s); EPI, epinephrine; FU, follow up; GA, gestational age; Gr, group(s); h, hour/s; MDI, Mental Development Index; mo, month; ND, neurodevelopment(al); NDI, neurodevelopmental impairment; PIVH, peri- intra-ventricular hemorrhage; PL, placebo; PMA, postmenstrual age; PVL, periventricular leukomalacia; RCT, randomized control trial; sign., significant; SVC, superior vena cava; TGr, treatment group; VLBWI, very low birth weight infants; WoL, week of life.

**Table 3:** Studies comparing the vasoactive medications used in neonates.

Compared Medications [References]	Author (Year)/Design	Population	Results & Authors’ Conclusions
DOP vs. PL [209]	DiSessa et al. (1981) [209]/RCT.	14 asphyxiated term neonates.	DOP raises AP and improve cardiac function in asphyxiated neonates.
DOP vs. PL [227]	Gilli et al. (1993) [227]/RCT.	39 VLBWI (<24 hol).	Response rate was higher in DOP. DOP treatment should be used earlier in hypotensive neonates.
DOP vs. DOB [56]	Roze et al. (1993) [56]/RCT.	20 hypotensive neonates (GA < 32 weeks) in first DoL.	DOP increased AP more efficiently, while only DOB increased the LVO.
DOP vs. DOB [228]	Klarr et al. (1994) [228]/RCT.	63 hypotensive neonates (GA < 35 weeks) in the first 24 hol.	DOP is more effective than DOB for the early treatment of AH.
DOP vs. DOB [58,60]	Osborn et al. (2002 and 2007) follow up at 3 Years [58,60]/RCT.	42 neonates (GA < 30 weeks) with low SVC flow in the first 24 hol.	DOB was more efficient in increasing blood flow, but less efficient in increasing AP. No dif. in long-term ND at 3 YoL.
DOP vs. DOB [10]	Lasky et al. (2011) [10]/retrospective cohort study.	287 LBWI < 1 mo of age.	No dif. in mortality between DOP and DOB. Treatment with DOP alone was more common.
DOP vs. DOB [116]	Filippi et al. (2007) [116]/non-blind RCT.	35 hypotensive VLBWI.	DOP is more effective than DOB in increasing systemic AP. DOP reduces TSH, T4, and prolactin.
DOP vs. DOB [16]	Subhedar et al. (2003) [16]/Cochrane review.	209 infants (GA 23–36 weeks) < 28 days.	DOP was more effective than DOB for short-term treatment. The long-term effect on ND is unknown.
DOP vs. DOB vs. EPI vs. NE vs. MIL vs. AVP vs. LEVO vs. corticosteroids vs. volume.	Sarafidis et al. (2022) [61]/Systematic review and pairwise meta-analysis.	19 studies in 758 hypotensive term and preterm neonates.	DOP more effectively increased AP than DOB.
DOP vs. EPI [65]	Pellicer et al. (2005) [65]/RCT.	59 hypotensive neonates (GA < 32 weeks) aged 2–16 h.	Both medications showed comparable increases in BP, cerebral oxygenation, CBF, response rate, and need for rescue therapy.
DOP vs. EPI [66]	Valverde et al. (2006) [66]/RCT.	60 hypotensive LBWI (GA < 32 weeks) < 24 hoL.	EPI is as effective as DOP for the treatment of AH in LBWI, but it is associated with more adverse effects.
DOP vs. EPI [59]	Pellicer et al. (2009) [59]/RCT.	130 LBWI (GA < 32 weeks) < 24 hoL.	Cautious use of CV support for early systemic AH in LBWI seems to be safe.
DOP vs. NE as 1st-line treatment [69]	Nissimov et al. (2023) [69]/retrospective study.	156 neonates (<35 weeks PMA) with sepsis or NEC.	NE was associated with decreased mortality, neurologic injury, and occurrence of NEC/sepsis among the survivors.
DOP vs. AVP [78]	Rios et al. (2015) [78]/RCT.	Hypotensive ELBWI (GA < 30 weeks) first 24 hoL.	AVP-Gr received fewer doses of surfactant, had lower PaCO2, and were not tachycardic.
DOP vs. Hydrocortisone [229]	Bourchier and Weston (1997) [229]/RCT.	40 hypotensive VLBWI (GA 27 weeks).	Successful treatment: hydrocortisone 81% vs. DOP 100%. No dif. in any clinical outcome.
DOB vs. PL [14,113]	Bravo et al. (2015 & 2021) [14,113]/RCT exploratory short-term outcome and long-term studies.	127 infants (GA < 31 weeks); 28 with low SVC flow and 98 normal SVC flow, within the first 24 hol.	SVC flow increased in the entire cohort. No dif. in AP and other clinical and biochemical parameters. No dif. in the combined outcome (mortality or NDI at 6 years).
DOB vs. MIL [149]	No study in neonates. Cavigelli-Brunner et al. (2018) [149]/pilot RCT in children.	50 children (age 2.5 mo to 14.2 years).	DOB and MIL are safe, well tolerated, and equally effective in prevention of LCOS after pediatric cardiac surgery.
EPI vs. no treatment vs. other inotropes [230]	Paradisis et al. (2004) [230]/Cochrane review.	No published study was found.	
EPI vs. hydrocortisone as adjuvant treatments [47]	Foote et al. (2023) [47]/multicenter RCT.	1592 infants with septic shock refractory to DOP.	The use of hydrocortisone as an adjuvant treatment was associated with decreased mortality. EPI alone or in combination therapy was associated with worse outcomes.
MIL vs. placebo [140]	Hoffman et al. (2003) [140]/RCT.	238 neonates and children (aged 2 days to 6.9 years) in high-risk for LCOS after corrective cardiac surgery.	High-doses of MIL reduces the risk of LCOS after cardiac surgery.
MIL vs. PL [145]	Paradisis et al. (2009) [145]/RCT.	90 infants (GA < 30 weeks; age < 6 h) in high risk of low SVC flow.	MIL did not prevent low systemic blood flow during the first 24 h. MIL had higher HR. No dif. in AP, inotrope use, PIVH, other clinical outcomes, and mortality or side effects.
MIL vs. no prophylaxis [136]	Halliday et al. (2017) [136]/Retrospective study.	45 preterm neonates (GA 23–26 weeks) subjected to PDA ligation.	MIL-Gr had higher AP. No dif. in inotrope and hydrocortisone use, or clinical outcomes. Prophylactic after PDA ligation does not sign. affect CV stability or long-term outcome.
MIL vs. LEVO [183]	Momeni et al. (2011) [183]/RCT.	36 infants and children (age range 7–977 d) operated for CHD.	LEVO-Gr had lower myocardial oxygen demands and troponin levels postoperatively. LEVO is at least as efficacious as MIL.
MIL vs. LEVO [150]	Lechner et al. (2012) [150]/RCT; prophylactic LEVO vs. MIL to prevent LCOS.	40 term infants undergoing repair of CHD.	Postoperative cardiac output and index were similar in LEVO vs. MIL. Improvement in cardiac function in the MIL-Gr. Both drugs were well tolerated; no death or serious adverse event.
MIL vs. LEVO [36]	Pellicer et al. (2013) [36]/RCT.	20 term neonates undergoing surgical repair for CHD.	MIL Gr had lower pH and higher blood glucose and inotrope score. Study drug withdrawal at 96 h was more frequent in LEVO-Gr. LEVO is well tolerated and may have advantages over MIL in terms of the dosing regimen.
LEVO vs. standard inotrope treatment [93]	Ricci et al. (2012) [93]/RCT.	63 neonates (<30 days) at risk of low SVC flow post-surgery for CHD.	The occurrence of LCOS, HR, lactate levels, and inotrope score were sign. lower in LEVO Gr. No dif. in mortality and clinical outcomes. LEVO was well tolerated with a potential benefit on postoperative hemodynamic and metabolic parameters.
LEVO vs. standard inotrope treatment [186]	Hummel et al. (2017) [186]/Cochrane.	Five RCTs with a total of 212 neonates and children under 5 years undergoing surgery for CHD.	LEVO showed no clear effect on mortality and clinical outcomes. Current evidence is insufficient to suggest LEVO for prevention of LCOS and mortality.
Hydrocortisone vs. placebo as rescue treatment [98]	Ng et al. (2006) [98]/RCT.	48 VLBW infants with refractory AH.	A stress dose of hydrocortisone was effective in treating refractory AH in VLBW infants. However, routine corticosteroids could not be recommended because of their potential adverse effects.
Hydrocortisone vs. placebo as rescue treatment [97]	Kovacs et al. (2019) [97]/RCT.	35 cooled asphyxiated term neonates with volume-resistant AH.	Hydrocortisone use effectively increased the AP and decreased the inotrope needs in cooled asphyxiated neonates with resistant AH.
Hydrocortisone vs. placebo [194]	Ando et al. (2005) [194]/RCT.	Twenty neonates (age < 28 days) undergoing biventricular repair.	Hydrocortisone improved hemodynamic profile and decreased the inotrope score without increasing the risk of complications. Adrenal insufficiency may occur after neonatal open-heart surgery.
Dexamethasone vs. placebo as adjuvant therapy [231]	Gaissmaier et al. (1999) [231]/RCT.	20 neonates (GA 25–36 vertical alignment) < 1 mo, who with inotrope-refractory AH.	DXM was effective for the management of severe AH in preterm infants not responding to standardized treatment.

AH, arterial hypotension; AP, arterial pressure; AVP, vasopressin; CHD, congenital heart disease; CV, cardiovascular; Dif., difference; DOB, dobutamine; DoL, day of life; DOP, dopamine; DXM, dexamethasone; ELBWI, extremely low birth weight infants; EPI, epinephrine; GA, gestational age; Gr, group(s); hol, hours of life; LBWI, low birth weight infants; LCOS, low cardiac output syndrome; LEVO, levosimendan; LVO, left ventricular output; MIL, milrinone; mo, month; ND, neurodevelopment; NDI, neurodevelopmental impairment; NE, norepinephrine; NEC, necrotizing enterocolitis; PIVH, peri-intra-ventricular hemorrhage; PL, placebo; PMA, postmenstrual age; RCT, randomized control trial; sign., significant; SVC, superior vena cava; VLBWI, very low birth weight infants; YoL, year(s) of life.

## Data Availability

Not applicable.

## Supplementary Materials

The following supporting information can be downloaded at: https://www.mdpi.com/article/10.3390/children11040490/s1, Table S1. Studies comparing the vasoactive medications used in neonates. Ref. [256] is cited in supplementary materials.
