# Supplementary material for: An Update on Pharmacologic Management of Neonatal Hypotension: When, Why, and Which Medication
Source: Children (Basel). 2024 Apr 19;11(4):490. doi: 10.3390/children11040490 (PMC11049273; doi:10.3390/children11040490)
Supplement: Supplementary file 1 [file children-11-00490-s001.zip › children-2914444-supplementary.pdf]

Supplementary Table

Table S1. Studies comparing the vasoactive medications used in neonates

Compared medications	First author (year) [Ref.] / design	Population	Main results	Authors' conclusions
DOP vs. PL (D/W)	DiSessa et al. (1981) [1] / RCT to compared DOP vs. PL.	14 severely asphyxiated term neonates (7 in each group)	AP increased and caECHO indices improved only in the DOP-GR.	Low doses of DOP raise AP and improve cardiac function in asphyxiated neonates.
DOP vs. PL (plasma protein fraction)	Gilli et al. (1993) [2] / RCT	39 hypotensive VLBWI aged < 24 h of NICU admission	Response rate was higher in DOP-Gr (89%) vs. PL-Gr (45%) (p <0.009)	DOP treatment should be used earlier in hypotensive neonates.
DOP vs. DOB	Roze et al. (1993) [3] / RCT	20 hypotensive neonates (GA< 32 wks) in the first postnatal days.	AP increased at a DOB dose of 20 mcg/kg/min and DOP dose of 12 mcg/kg/min. The LVO increased (+21%) with DOB and decreased with DOP (-14%).	DOP increased and maintained AP more efficiently than DOB, while only DOB increased the LVO.
DOP vs. DOB	Klarr et al. (1994) [4] / RCT	63 hypotensive neonates (GA <35 wks) in the first 24 hours of life.	DOP at <10 more effectively increased AP than DOB (successful rate 100% vs. 84%).	DOP is more effective than DOB for the early treatment of AH.
DOP vs. DOB	Osborn et al. (2002) short-term study [5] & Osborn et al. (2007) follow up at 3 Year [6] / RCT	42 neonates (GA<30 wks) with low SVC flow within 24 hours after birth. DOP-Gr n= 20, DOB-Gr n= 22.	DOP induced a greater increase in AP; DOB induced higher increase in SVC flow; no difference in mortality, morbidity, and combined death and disability at 3 years.	Compared to DOP, DOB was more efficient in increasing blood flow, but less efficient in increasing AP. No dif. In in long-term outcome.
DOP vs. DOB	Lasky et al. (2011) [7] / multicenter retrospective cohort study.	287 LBWI < 1 mo of age. DOP alone, n 194; DOB alone, n=14; both DOP & DOB, n=79.	In-hospital mortality: 18.2% vs. 20% vs. 46.4%, respectively (P =0.004).	No dif. In mortality between DOP and DOB. Treatment with DOP alone was more common.
DOP vs. DOB	Filippi et al. (2007) [8] / non-blind RCT	35 hypotensive VLBWI (Birth weight < 1500 g). DOP-Gr n=18; DOB-Gr n=17.	The dosage to normalize AP was sign. Higher for DOB. DOP, but not DOB, was assoc. with suppression of TSH, T4, and prolactin.	DOP is more effective than DOB in increasing systemic AP. DOP reduces transiently levels of TSH, T4, and prolactin.
DOP vs. DOB	Subhedar et al. (2003) [9] / Cochrane review of 5 RCTs.	209 infants (GA 23-36 wks) treated with inotropes at < 28 days postnatal age; n= 104 and 105 in DOP and DOB Gr, respectively.	DOP was more effective in treating systemic AH. No dif. In neonatal mortality and short-term clinical outcomes.	DOP was more effective than DOB for short-term treatment. The long-term effect on ND is unknown.
Treatments evaluated: DOP, DOB, EPI, NE, MIL, AVP, LEVO, corticosteroids, and volume therapy.	Sarafidis et al. (2022) [10] / Systematic review and pairwise meta-analysis.	19 studies in 758 hypotensive term and preterm neonates receiving anti-hypotensive treatments.	DOP was almost 3 times more effective than DOB and 10 – 23 times more effective than volume/PL in normalizing AP.	DOP more effectively increased AP than DOB. The low number of RCTs concerning other agents did not allow any firm conclusion to be reached.

Compared medications	First author (year) [Ref.] / design	Population	Main results	Authors' conclusions
DOP vs. EPI	Pellicer et al. (2005) [11] / RCT to explore the effects of DOP vs. EPI on brain hemodynamics	59 hypotensive neonates (GA <32 wks) aged 2-16 h: 32 in EPI-Gr and 27 in DOP-Gr.	AP increased in comparable proportion of the DOP and EPI groups. No dif. In AP, cerebral oxygenation and CBF. The EPI Gr had higher HR, blood glucose, and need for insulin.	Both medications induced comparable (a) increases in BP, cerebral oxygenation, and CBF; and (b) response rate and need for rescue therapy.
DOP vs. EPI	Valverde et al. (2006) [12] / RCT to compare the effects of low/moderate-dose DOP vs. EPI on AP, clinical outcomes, and adverse effects.	60 hypotensive LBWI (GA <32wks) < 24 hoL. 2 Grs: DOP-Gr n=28; EPI-Gr n=32.	No sign. Dif. In AP increase and treatment failure or need for rescue therapy. EPI-Gr had higher HR, blood lactate and glycose, lower base excess and increased need for insulin.	Low/moderate-dose of EPI is as effective as low/moderate-dose of DOP for the treatment of AH in LBWI, but it is associated with more adverse effects.
DOP vs. EPI	Pellicer et al. (2009) [13] / RCT exploring the effect of DOP vs. EPI for early AH on ND.	130 LBWI (GA <32wks) < 24 hoL. Hypotensive (TGr) n= 60 (28 in DOP-Gr & 32 in EPI-Gr); normotensive (CGr) n=70.	No sign. Dif. Between DOP and EPI in the rates of combined adverse outcome (death or CP or severe NDI)	Cautious use of CV support for early systemic AH in LBWI seems to be safe.
DOP vs. NE	Nissimov et al. (2023) [14] / retrospective study to compare the clinical effectiveness of DOP vs. NE as first-line therapy for sepsis-related hypotension.	156 neonates (<35 weeks PMA) with sepsis or NEC treated with DOP (n=113) or NE (n=43) as primary therapy for hypotension.	NE was associated with lower mortality and decreased neurologic injury and occurrence of NEC/sepsis among the survivors.	NE may be more effective than DOP for management of sepsis-related hypotension among preterm infants.
DOP vs. AVP	Rios et al. (2015) [15] / RCT.<br>AIM: To evaluate AVP vs DOP as initial therapy in ELBWI with hypotension.	Hypotensive ELBWI (GA <30 wks) during the first 24 hours after birth. 2 Grs: DOP-Gr (n=10) and AVP-Gr (n=10)	AP increased by 90% in both Grs. Compared to DOP-Gr, the AVP-Gr received fewer doses of surfactant, had lower PaCO2, and were not tachycardic	AVP in ELBW infants as the initial agent for early hypotension appeared to be safe.
DOP vs. Hydrocortisone	Bourchier and Weston (1997) [16] / RCT comparing the efficacy between hydrocortisone and DOP for treatment of AH.	40 hypotensive VLBWI (GA 27 wks) requiring inotropic support within 24 hours of birth. 2 Grs:<br>Hydrocortisone-Gr (n=21) and DOP-Gr (n=19).	Successful treatment:<br>hydrocortisone 81% vs. DOP 100%. No dif. In any clinical outcome.	Both DOP and hydrocortisone are effective treatments for hypotension in VLBWI.
DOB vs. PL	Bravo et al. (2015 & 2021) [17,18] / RCT exploratory short-term outcome & long-term studies / AIM: To evaluate the effectiveness of DOB vs. PL for treating LSVC flow and long-term outcomes.	127 infants (GA <31 wks); 28 with low SVC flow and 98 normal SVC flow, within the first 24 hours after birth. The low flow Gr was randomly assigned to the DOB-Gr (n- 16) and PL-Gr (n=12).	SVC flow increased in the entire cohort and 26/28 of randomized infants. No dif. In AP and other clinical and biochemical parameters. The DOB-Gr showed higher HR and improved base excess.	There was a tendency toward improved short-term clinical and biochemical data in infants with low SVC flow treated with DOB.<br>No dif. In the combined outcome (mortality or NDI) between DOP and PL Grs at 6 years.
DOB vs. MIL	No study in neonates.<br>Cavigelli-Brunner et al. (2018) [19] / pilot RCT in children to assess the efficacy of	50 children (age 2.5 mo to 14.2 years) treated with either DOB (n=26) or MIL	A trend towards higher systolic AP in DOB Gr. Both drugs increased HR. No dif. In clinical outcomes. Both drugs were well tolerated.	DOB and MIL are safe, well tolerated, and equally effective in prevention of LCOS after pediatric cardiac surgery.

Compared medications	First author (year) [Ref.] / design	Population	Main results	Authors' conclusions
	DOB vs. MIL in preventing LCOS.	(n=24) for the first 36 postoperative hours.		
EPI vs. no treatment	Paradisis et al. (2004) [20] / Cochrane review to comparing EPI vs. no treatment or other inotropes.	No published study was found.		
EPI vs. hydrocortisone as adjuvant treatments	Foote et al. (2023) [21] / multicenter RCT to compare EPI vs. hydrocortisone as rescue treatment.	1592 infants with septic shock refractory to DOP receiving EPI or Hydrocortisone as adjuvant treatment.	Compared to DOP alone, mortality rate increased sig. after addition of EPI and decreased when hydrocortisone was added.	The use of hydrocortisone as an adjuvant treatment was associated with decreased mortality. EPI alone or in combination therapy was associated with worse outcomes.
MIL vs. placebo	Hoffman et al. (2003) [22] / RCT comparing the effect of MIL vs. PL in preventing LCOS.	Neonates and children (aged 2 days to 6.9 years) in high-risk for LCOS-after corrective cardiac surgery.	MIL sign. Reduced the risk of LCOS.	High-doses of MIL reduces the risk of LCOS after cardiac surgery.
MIL vs. PL	Paradisis et al. (2009) [23] / RCT to assess the effectiveness of MIL vs. PL for prevention of low systemic blood flow in high-risk preterm infants.	90 infants (GA <30 wks; age < 6 h) in high risk of low SVC flow. 2 groups: MIL n=42, PL n=48.	Low SVC flow prevention: 83% vs. 81% in MIL vs. PL. No dif. In AP, inotrope use, PIVH, other clinical outcomes, and mortality or side effects. MIL-Gr had higher HR.	MIL did not prevent low systemic blood flow during the first 24 hours in high-risk preterm infants. MIL had no adverse effects.
MIL vs. no prophylaxis	Halliday et al. (2017) [24] / Retrospective study to explore the effect of prophylactic MIL on CV stability and long-term outcomes.	45 preterm neonates (GA 23-26 wks) receiving PDA ligation. 2 groups: MIL-Gr n=15 receiving prophylactic MIL; CGr (no prophylaxis), n=30.	MIL-Gr had higher AP than the PL-Gr at 18 – 24 h after surgery. No dif. In inotrope and hydrocortisone use, or clinical outcomes.	Prophylactic MIL use in VLBWI after PDA ligation does not sign. Affect CV stability or long-term outcome.
MIL vs. LEVO	Momeni et al. [25] (2011) / RCT to compare the effect of LEVO vs. MIL on hemodynamic and biochemical parameters after surgery for CHD.	36 infants and children (age range 7 – 977 d) operated for CHD who received EPI, were randomized to receive MIL or LEVO as adjuvant treatment.	The LEVO-Gr had sign. Lower myocardial oxygen demands and a trend towards lower troponin levels postoperatively.	LEVO is at least as efficacious as MIL after corrective surgery for CHD.
MIL vs. LEVO	Lechner et al. [26] (2012) / RCT to compare the effectiveness of LEVO vs. MIL to prevent LCOS after corrective open-heart surgery.	40 term infants undergoing repair of CHD were randomized to receive either MIL (n=20) or LEVO (n=20).	No diff. in hemodynamic profile. Only the LEVO-Gr showed an increase in cardiac output and cardiac index over time. Both drugs were well tolerated; no death or serious adverse event occurred.	Postoperative cardiac output and index were similar in neonates receiving prophylactic either LEVO or MIL. An improvement of cardiac function over-time was observed in the MIL-Gr.
MIL vs. LEVO	Pellicer et al. [27] (2013) / RCT to examine the efficacy and safety of MIL vs. LEVO in neonates undergoing CV surgery.	20 term neonates undergoing surgical repair for CHD received MIL (n=9) or LEVO (n=11).	A time-related, group-independent increase in cerebral oxygenation and decrease in diastolic AP was observed post-surgery. MIL group had lower pH and higher blood glucose and inotrope score. Study drug withdrawal at 96 h was more frequent in LEVO-Gr.	LEVO is well tolerated in critically ill neonates and it may have advantages over MIL in terms of the dosing regimen.

Compared medications	First author (year) [Ref.] / design	Population	Main results	Authors' conclusions
LEVO vs. standard inotrope treatment	Ricci et al. (2012) [28] / RCT to evaluate safety and efficacy of LEVO vs. standard treatment in neonates with CHD undergoing cardiac surgery.	63 neonates (< 30 days) at risk of low SVC flow post-surgery for CHD; 32 cases and 31 controls who received LEVO or standard inotrope, respectively.	The occurrence of LCOS, HR, lactate levels, and inotrope score were sign. Lower in the LEVO-Gr. No sign. Diff. in mortality and duration of mechanical ventilation and stay in pediatric cardiac intensive care unit.	LEVO infused in neonates undergoing cardiac surgery was well tolerated with a potential benefit on postoperative hemodynamic and metabolic parameters.
LEVO vs. standard inotrope treatment	Hummel et al. (2017) [29] / Cochrane review to evaluate the efficacy and safety of the postoperative use of LEVO for LCOS prevention.	Five RCTs with a total of 212 neonates and children under 5 years undergoing surgery for CHD that received prophylactic LEVO or standard inotrope treatment.	LEVO showed no clear effect on mortality risk compared to standard treatments. No difference in clinical outcomes.	Current evidence is insufficient to suggest LEVO administration for prevention of LCOS and mortality post-cardiac surgery.
Hydrocortisone vs. placebo as rescue treatment	Ng et al. (2006) [30] / RCT to assess the effectiveness of a "stress dose" of hydrocortisone for rescue treatment of DOP refractory hypotension and adrenocortical insufficiency of prematurity.	48 VLBW infants with refractory AH received either a stress dose of hydrocortisone (n=24) or PL (N/S, n= 24).	Hydrocortisone-Gr had sign. higher AP and shorter duration of vasopressor support compared to PL-Gr.	A stress dose of hydrocortisone was effective in treating refractory AH in VLBW infants. However, routine and prophylactic use of systemic corticosteroids could not be recommended because of their potential adverse effects.
Hydrocortisone vs. placebo as rescue treatment	Kovacs et al. (2019) [31] / RCT to examine whether hydrocortisone increases AP and decreases inotrope requirements compared with PL in cooled, asphyxiated neonates with volume-resistant hypotension.	35 asphyxiated term neonates with volume-resistant hypotension received hydrocortisone or placebo in addition to standard dopamine treatment during hypothermia.	More neonates of the hydrocortisone Gr reached the target MAP; duration of CV support and inotrope dosage were lower in the hydrocortisone Gr.	Hydrocortisone use effectively increased the AP and decreased the inotrope needs in cooled asphyxiated neonates with resistant hypotension.
Hydrocortisone vs. placebo	Ando et al. (2005) [32] / RCT to investigate (a) whether adrenal insufficiency exists after cardiopulmonary bypass in neonates and (b) assess the legitimacy of the routine steroid use for this patient cohort.	Twenty neonates (age < 28 days) undergoing biventricular repair: 10 received hydrocortisone and 10 received PL.	Hydrocortisone improved hemodynamic profile and increased the inotrope score without increasing the risk of infection, peptic ulcer, or pituitary–adrenal suppression.	Adrenal insufficiency may occur after neonatal open-heart surgery. Stress-dose hydrocortisone blunts other organ dysfunction without increasing the risk of complications.
Dexamethsone vs. placebo as adjuvant therapy	Gaissmaier et al. (1999) [33] / RCT to test the efficacy of single-dose DXM in the management of inotrope refractory AH of neonates.	20 hypotensive neonates (GA 25-36 wks) < 1mo, who did not respond to a standardized inotrope treatment were started on EPI and were randomly allocated to receive either DXM or PL.	EPI infusion stopped in 5/8 infants in DXM Gr but in only 1 of 9 infants in the PL-Gr. The duration of EPI was sign. Shorter in the DXM Gr.	DXM was effective for the management of severe AH in preterm infants not responding to standardized treatment.

AH, arterial hypotension; AP, arterial pressure; Assoc., association/ted; AVP, vasopressin; caECHO, echocardiography; CGr, control group; CP, cerebreal palcy; CV, cardiovascular; Dif. Difference; DOB, dobutamine; DoL, day of life; DOP, dopamine; DXM, dexamethasone; EPI, epinephrine; Ga, gestational age; Gr, group(s); LBWI, low birth weight infants; LCOS, low cardiac output syndrome; LEVO, levosimendan; LVO, left ventricular output; MIL, milrinone; mo, month; NDI, neurodevelopmental impairment; NE, norepinephrine; PL, placebo; PMA, postmenstrual age; RCT, randomized control trial; sign., significant; SVC, superior vena cava; TGr, treatment group; VLBWI, very low birth weight infants; wks, weeks.

References

1. DiSessa, T.G.; Leitner, M.; Ti, C.C.; Gluck, L.; Coen, R.; Friedman, W.F. The Cardiovascular Effects of Dopamine in the Severely Asphyxiated Neonate. *J Pediatr* **1981**, *99*, 772–776, doi:10.1016/s0022-3476(81)80409-x.
2. Gill, A.B.; Weindling, A.M. Randomised Controlled Trial of Plasma Protein Fraction versus Dopamine in Hypotensive Very Low Birthweight Infants. *Arch Dis Child* **1993**, *69*, 284–287, doi:10.1136/adc.69.3_spec_no.284.
3. Rozé, J.C.; Tohier, C.; Maingueneau, C.; Lefèvre, M.; Mouzard, A. Response to Dobutamine and Dopamine in the Hypotensive Very Preterm Infant. *Arch Dis Child* **1993**, *69*, 59–63, doi:10.1136/adc.69.1_spec_no.59.
4. Klarr, J.M.; Faix, R.G.; Pryce, C.J.; Bhatt-Mehta, V. Randomized, Blind Trial of Dopamine versus Dobutamine for Treatment of Hypotension in Preterm Infants with Respiratory Distress Syndrome. *J Pediatr* **1994**, *125*, 117–122, doi:10.1016/s0022-3476(94)70137-7.
5. Osborn, D.; Evans, N.; Kluckow, M. Randomized Trial of Dobutamine versus Dopamine in Preterm Infants with Low Systemic Blood Flow. *J Pediatr* **2002**, *140*, 183–191, doi:10.1067/mpd.2002.120834.
6. Osborn, D.A.; Evans, N.; Kluckow, M.; Bowen, J.R.; Rieger, I. Low Superior Vena Cava Flow and Effect of Inotropes on Neurodevelopment to 3 Years in Preterm Infants. *Pediatrics* **2007**, *120*, 372–380, doi:10.1542/peds.2006-3398.
7. Lasky, T.; Greenspan, J.; Ernst, F.R.; Gonzalez, L. Dopamine and Dobutamine Use in Preterm or Low Birth Weight Neonates in the Premier 2008 Database. *Clin Ther* **2011**, *33*, 2082–2088, doi:10.1016/j.clinthera.2011.11.001.
8. Filippi, L.; Pezzati, M.; Poggi, C.; Rossi, S.; Cecchi, A.; Santoro, C. Dopamine versus Dobutamine in Very Low Birthweight Infants: Endocrine Effects. *Arch Dis Child Fetal Neonatal Ed* **2007**, *92*, F367-371, doi:10.1136/adc.2006.098566.
9. Subhedar, N.V. Treatment of Hypotension in Newborns. *Semin Neonatol* **2003**, *8*, 413–423, doi:10.1016/S1084-2756(03)00117-9.
10. Sarafidis, K.; Verykouki, E.; Nikopoulos, S.; Apostolidou-Kiouti, F.; Diakonidis, T.; Agakidou, E.; Kontou, A.; Haidich, A.-B. Systematic Review and Meta-Analysis of Cardiovascular Medications in Neonatal Hypotension. *Biomed Hub* **2022**, *7*, 70–79, doi:10.1159/000525133.
11. Pellicer, A.; Valverde, E.; Elorza, M.D.; Madero, R.; Gayá, F.; Quero, J.; Cabañas, F. Cardiovascular Support for Low Birth Weight Infants and Cerebral Hemodynamics: A Randomized, Blinded, Clinical Trial. *Pediatrics* **2005**, *115*, 1501–1512, doi:10.1542/peds.2004-1396.

12. Valverde, E.; Pellicer, A.; Madero, R.; Elorza, D.; Quero, J.; Cabañas, F. Dopamine versus Epinephrine for Cardiovascular Support in Low Birth Weight Infants: Analysis of Systemic Effects and Neonatal Clinical Outcomes. *Pediatrics* **2006**, *117*, e1213-1222, doi:10.1542/peds.2005-2108.
13. Pellicer, A.; Bravo, M. del C.; Madero, R.; Salas, S.; Quero, J.; Cabañas, F. Early Systemic Hypotension and Vasopressor Support in Low Birth Weight Infants: Impact on Neurodevelopment. *Pediatrics* **2009**, *123*, 1369–1376, doi:10.1542/peds.2008-0673.
14. Nissimov, S.; Joye, S.; Kharrat, A.; Zhu, F.; Ripstein, G.; Baczynski, M.; Choudhury, J.; Jasani, B.; Deshpande, P.; Ye, X.Y.; et al. Dopamine or Norepinephrine for Sepsis-Related Hypotension in Preterm Infants: A Retrospective Cohort Study. *Eur J Pediatr* **2023**, *182*, 1029–1038, doi:10.1007/s00431-022-04758-4.
15. Rios, D.R.; Kaiser, J.R. Vasopressin versus Dopamine for Treatment of Hypotension in Extremely Low Birth Weight Infants: A Randomized, Blinded Pilot Study. *J Pediatr* **2015**, *166*, 850–855, doi:10.1016/j.jpeds.2014.12.027.
16. Bourchier, D.; Weston, P.J. Randomised Trial of Dopamine Compared with Hydrocortisone for the Treatment of Hypotensive Very Low Birthweight Infants. *Arch Dis Child Fetal Neonatal Ed* **1997**, *76*, F174-178, doi:10.1136/fn.76.3.f174.
17. Bravo, M.C.; López-Ortego, P.; Sánchez, L.; Riera, J.; Madero, R.; Cabañas, F.; Pellicer, A. Randomized, Placebo-Controlled Trial of Dobutamine for Low Superior Vena Cava Flow in Infants. *J Pediatr* **2015**, *167*, 572-578.e1-2, doi:10.1016/j.jpeds.2015.05.037.
18. Bravo, M.C.; López-Ortego, P.; Sánchez, L.; Díez, J.; Cabañas, F.; Pellicer, A. Randomised Trial of Dobutamine versus Placebo for Low Superior Vena Cava Flow in Preterm Infants: Long-Term Neurodevelopmental Outcome. *J Paediatr Child Health* **2021**, *57*, 872–876, doi:10.1111/jpc.15344.
19. Cavigelli-Brunner, A.; Hug, M.I.; Dave, H.; Baenziger, O.; Buerki, C.; Bettex, D.; Cannizzaro, V.; Balmer, C. Prevention of Low Cardiac Output Syndrome After Pediatric Cardiac Surgery: A Double-Blind Randomized Clinical Pilot Study Comparing Dobutamine and Milrinone. *Pediatr Crit Care Med* **2018**, *19*, 619–625, doi:10.1097/PCC.0000000000001533.
20. Paradisis, M.; Osborn, D.A. Adrenaline for Prevention of Morbidity and Mortality in Preterm Infants with Cardiovascular Compromise. *Cochrane Database Syst Rev* **2004**, CD003958, doi:10.1002/14651858.CD003958.pub2.
21. Foote, H.P.; Benjamin, D.K.; Greenberg, R.G.; Clark, R.H.; Hornik, C.P. Use of Vasopressors for Septic Shock in the Neonatal Intensive Care Unit. *J Perinatol* **2023**, *43*, 1274–1280, doi:10.1038/s41372-023-01667-8.
22. Hoffman, T.M.; Wernovsky, G.; Atz, A.M.; Kulik, T.J.; Nelson, D.P.; Chang, A.C.; Bailey, J.M.; Akbary, A.; Kocsis, J.F.; Kaczmarek, R.; et al. Efficacy and Safety of Milrinone in Preventing Low Cardiac Output Syndrome in Infants and Children after Corrective Surgery for Congenital Heart Disease. *Circulation* **2003**, *107*, 996–1002, doi:10.1161/01.cir.0000051365.81920.28.
23. Paradisis, M.; Evans, N.; Kluckow, M.; Osborn, D. Randomized Trial of Milrinone versus Placebo for Prevention of Low Systemic Blood Flow in Very Preterm Infants. *J Pediatr* **2009**, *154*, 189–195, doi:10.1016/j.jpeds.2008.07.059.
24. Halliday, M.; Kavarana, M.; Ebeling, M.; Kiger, J. Milrinone Use for Hemodynamic Instability in Patent Ductus Arteriosus Ligation. *J Matern Fetal Neonatal Med* **2017**, *30*, 529–533, doi:10.1080/14767058.2016.1177720.
25. Momeni, M.; Rubay, J.; Matta, A.; Rennotte, M.-T.; Veyckemans, F.; Poncelet, A.J.; Clement de Clety, S.; Anslot, C.; Joomye, R.; Detaille, T. Levosimendan in

Congenital Cardiac Surgery: A Randomized, Double-Blind Clinical Trial. *J Cardiothorac Vasc Anesth* **2011**, *25*, 419–424, doi:10.1053/j.jvca.2010.07.004.
26. Lechner, E.; Hofer, A.; Leitner-Peneder, G.; Freynschlag, R.; Mair, R.; Weinzettel, R.; Rehak, P.; Gombotz, H. Levosimendan versus Milrinone in Neonates and Infants after Corrective Open-Heart Surgery: A Pilot Study. *Pediatr Crit Care Med* **2012**, *13*, 542–548, doi:10.1097/PCC.0b013e3182455571.
27. Pellicer, A.; Riera, J.; Lopez-Ortego, P.; Bravo, M.C.; Madero, R.; Perez-Rodriguez, J.; Labrandero, C.; Quero, J.; Buño, A.; Castro, L.; et al. Phase 1 Study of Two Inodilators in Neonates Undergoing Cardiovascular Surgery. *Pediatr Res* **2013**, *73*, 95–103, doi:10.1038/pr.2012.154.
28. Ricci, Z.; Garisto, C.; Favia, I.; Vitale, V.; Di Chiara, L.; Cogo, P.E. Levosimendan Infusion in Newborns after Corrective Surgery for Congenital Heart Disease: Randomized Controlled Trial. *Intensive Care Med* **2012**, *38*, 1198–1204, doi:10.1007/s00134-012-2564-6.
29. Hummel, J.; Rücker, G.; Stiller, B. Prophylactic Levosimendan for the Prevention of Low Cardiac Output Syndrome and Mortality in Paediatric Patients Undergoing Surgery for Congenital Heart Disease. *Cochrane Database Syst Rev* **2017**, *3*, CD011312, doi:10.1002/14651858.CD011312.pub2.
30. Ng, P.C.; Lee, C.H.; Bnur, F.L.; Chan, I.H.S.; Lee, A.W.Y.; Wong, E.; Chan, H.B.; Lam, C.W.K.; Lee, B.S.C.; Fok, T.F. A Double-Blind, Randomized, Controlled Study of a “Stress Dose” of Hydrocortisone for Rescue Treatment of Refractory Hypotension in Preterm Infants. *Pediatrics* **2006**, *117*, 367–375, doi:10.1542/peds.2005-0869.
31. Kovacs, K.; Szakmar, E.; Meder, U.; Szakacs, L.; Cseko, A.; Vatai, B.; Szabo, A.J.; McNamara, P.J.; Szabo, M.; Jermendy, A. A Randomized Controlled Study of Low-Dose Hydrocortisone Versus Placebo in Dopamine-Treated Hypotensive Neonates Undergoing Hypothermia Treatment for Hypoxic-Ischemic Encephalopathy. *J Pediatr* **2019**, *211*, 13-19.e3, doi:10.1016/j.jpeds.2019.04.008.
32. Ando, M.; Park, I.-S.; Wada, N.; Takahashi, Y. Steroid Supplementation: A Legitimate Pharmacotherapy after Neonatal Open Heart Surgery. *Ann Thorac Surg* **2005**, *80*, 1672–1678; discusison 1678, doi:10.1016/j.athoracsur.2005.04.035.
33. Gaissmaier, R.E.; Pohlandt, F. Single-Dose Dexamethasone Treatment of Hypotension in Preterm Infants. *J Pediatr* **1999**, *134*, 701–705, doi:10.1016/s0022-3476(99)70284-2.
